# Aerogel-Based Single-Ion Magnets: A Case Study of a Cobalt(II) Complex Immobilized in Silica

**DOI:** 10.3390/molecules28010418

**Published:** 2023-01-03

**Authors:** Sergey Yu. Kottsov, Maxim A. Shmelev, Alexander E. Baranchikov, Mikhail A. Kiskin, Alim U. Sharipov, Nikolay N. Efimov, Irina K. Rubtsova, Stanislav A. Nikolaevskii, Gennady P. Kopitsa, Tamara V. Khamova, Ilya V. Roslyakov, Igor L. Eremenko, Vladimir K. Ivanov

**Affiliations:** 1 Kurnakov Institute of General and Inorganic Chemistry, Russian Academy of Sciences, Moscow 119991, Russia; 2 Faculty of Technology of Inorganic Substances and High Temperature Materials, Mendeleev University of Chemical Technology of Russia, Moscow 125047, Russia; 3 St.-Petersburg Nuclear Physics Institute, National Research Center “Kurchatov Institute”, Gatchina 188300, Russia; 4 Grebenshchikov Institute of Silicate Chemistry, Russian Academy of Sciences, St.-Petersburg 199034, Russia; 5 Department of Materials Science, Lomonosov Moscow State University, Moscow 119991, Russia; 6 Faculty of Chemistry, National Research University Higher School of Economics, Moscow 109028, Russia

**Keywords:** magnetic properties, aerogel, chemical immobilization, N-3-(trimethoxysilyl)propyl ethylenediamine, single-ion magnet, single-molecule magnet, Raman spectroscopy, electron paramagnetic resonance

## Abstract

The chemical immobilization of cobalt(II) ions in a silica aerogel matrix enabled the synthesis of the first representative example of aerogel-based single-ion magnets. For the synthesis of the lyogels, methyl-trimethoxysilane and N-3-(trimethoxysilyl)propyl ethylenediamine were co-hydrolyzed, then the ethylenediamine groups that were immobilized on the silica matrix enabled the subsequent binding of cobalt(II) ions. Lyogels with various amounts of ethylenediamine moieties (0.1–15 mol %) were soaked in isopropanol solutions of cobalt(II) nitrate and further supercritically dried in carbon dioxide to obtain aerogels with a specific surface area of 210–596 m^2^·g^−1^, an apparent density of 0.403–0.740 cm^3^·g^−1^ and a porosity of 60–78%. The actual cobalt content in the aerogels was 0.01–1.50 mmol per 1 g of SiO_2_, which could easily be tuned by the concentration of ethylenediamine moieties in the silica matrix. The introduction of cobalt(II) ions into the ethylenediamine-modified silica aerogel promoted the stability of the diamine moieties at the supercritical drying stage. The molecular prototype of the immobilized cobalt(II) complex, bearing one ethylenediamine ligand [Co(en)(MeCN)(NO_3_)_2_], was synthesized and structurally characterized. Using magnetometry in the DC mode, it was shown that cobalt(II)-modified silica aerogels exhibited slow magnetic relaxation in a nonzero field. A decrease in cobalt(II) concentration in aerogels from 1.5 mmol to 0.14 mmol per 1 g of SiO_2_ resulted in a weakening of inter-ion interactions; the magnetization reversal energy barrier likewise increased from 4 to 18 K.

## 1. Introduction

New magnetic materials are in great demand for the creation of new devices for high-density information storage, high-speed recording and processing, high-performance computing, and sensing or catalytic applications [1,2,3,4]. Among other materials, single-molecule and single-ion magnets (SMM or SIM) have attracted a great deal of research attention due to their unique advantages in the fields of molecular spintronics, molecular refrigeration, optical imaging, quantum computing, etc. [5,6]. In SIMs, the magnetization is stored within single ions, due to the very slow relaxation of their magnetic moments [7]. In order to avoid a magnetic exchange between paramagnetic ions, the ions must be spatially separated by a diamagnetic medium, thus resulting in magnetic dilution [8,9].

To date, the best performance in terms of the magnetization energy barrier *U*_eff_ and relaxation time *τ* has been achieved with lanthanide-based SIMs. Most of these, however, have little ability to withstand air and moisture [10]. Because of this issue, attention continues to focus on SIMs based on 3d transition metal complexes [11]. Among the 3d metals, cobalt is one of the most attractive candidates for the design of SIMs, as Co(II) exhibits a large spin-orbit coupling capability and its flexible coordination environment provides unique opportunities for the design of complexes possessing various geometries [12,13].

Recently, metal–organic frameworks (MOFs) have been recognized as an excellent platform on which to develop SIM materials, combining a well-controlled porous structure with a chemical flexibility that enables the introduction of magnetic complexes into the diamagnetic matrix [14]. MOF-based SIMs open up new possibilities for the design of multifunctional materials, as the magnetic ions in their structure selectively interact with paramagnetic molecules that enter the pores in the crystal [15]. For example, oxygen-responsive porous magnets for the non-cryogenic separation of molecular oxygen from air have been created using layered ruthenium complexes [16]. Other examples of SIM MOFs include rare earths [17,18,19], Mn_12_ clusters [14,20,21], and cobalt-based MOFs [22,23]. Among them, Co(II)-containing highly porous MOFs have been shown to have excellent sensitivity to various aromatic molecules, including thianthrene, toluene, and pyrrole [24], and can be used as selective sensors for dimethylformamide and water, and as CO_2_ storage materials [25].

The synthesis of MOFs is generally rather complicated; a further drawback is that MOFs can only be obtained as fine powders, which limits their use in the development of magnetic, sensor, or storage devices for practical applications. To solve this issue, MOFs can be encapsulated in a bulk matrix, e.g., polymeric, etc. [26]. Another advantageous route to creating highly porous SIMs is their incorporation in a sol-gel-derived diamagnetic matrix. The sol-gel approach provides a high level of chemical flexibility, due to the diversity of host matrices and the variability of synthetic parameters (solvent type, pH, various surfactants and chemical modifiers, aging techniques, post-synthesis treatment, etc.), which enables the synthesis of a wide range of materials with a precisely tunable structure, porosity, and other properties [27,28,29,30,31,32]. Comprehensive reviews of sol-gel-derived magnetic materials (but not specifically on SIMs or SMMs) have been published elsewhere [33,34].

Despite the prominence of the sol-gel approach for materials design, examples of sol-gel-derived SIMs or SMMs are scarce, and most of them are focused on the metal complexes immobilized in ordered mesoporous silica [35,36,37]. For example, Laskowska et al. incorporated Mn_12_ stearate clusters into ordered silica [38]. Solomon et al. also seized the challenge of creating disordered sol-gel SIMs by successfully incorporating a polyoxometalate magnetic iron-tungsten complex in a gelatin matrix [39]. Interestingly, the use of ordered matrices for the creation of SIMs was criticized by Pardo et al. [40]. In their pioneering work, Pardo et al. compared the state of nickel guest complexes, immobilized in both an ordered porous silica matrix and disordered silica xerogel. Although all the composites exhibited the properties of SMMs, with an increased superparamagnetic blocking temperature (*T*_b_ 4.5–10 K), on the surface of the ordered silica matrix, nickel complexes tended to form various oligomers, resulting in spin-glass behavior, while, in the xerogel host, only isolated magnetic clusters were found [40].

An example of very promising sol-gel-derived materials is aerogels with a three-dimensional open porous network of nanoparticles, exhibiting extremely high micro- and mesoporosity (even higher than 99%), a specific surface area of up to 1000 m^2^·g^−1^, and pore volumes > 1 cm^3^·g^−1^ [41]. The most striking feature of aerogels is their rich chemistry, which enables their surface immobilization with a wide variety of metal coordination compounds for the design of various functional materials, which might be luminescent, photochromic, etc. [42,43,44,45,46]. Among other aerogels, magnetic aerogels have attracted considerable interest [47]. The majority of them have been modified with magnetite (Fe_3_O_4_) nanoparticles to enable the preparation of efficient and easy-to-remove sorbents for spilled oil, dyes, and other environmental pollutants [48,49]. Similar materials have been reported to act as catalysts and in drug delivery or energy storage systems, etc. [50,51]. The immobilization of Fe_3_O_4_ nanoparticles onto a graphene aerogel surface resulted in an excellent electromagnetic shielding material [52]. Interestingly, aerogels can even be constructed from magnetic nanoparticles alone, e.g., Fe_1.3_Ni_0.7_P [9] or Fe_3_O_4_ [53].

Surprisingly, aerogel-based SIMs or SMMs have not yet been reported, and even aerogels that are modified with magnetic metal complexes have very rarely been mentioned. Khalf-Alla et al. immobilized square planar Pd(II) and octahedral Cu(II) complexes in starch and carboxymethyl cellulose aerogels, to produce antimicrobial and anti-cancer materials [54]. He et al. reported iron- and nitrogen-codoped carbon nanocomposites with single-copper sites to reduce the magnetic moment of iron centers and to enhance the electrocatalytic performance of the material [55].

This paper reports on the synthesis of amorphous silica aerogel monoliths that are modified with cobalt(II) ions to create single-ion mesoporous magnetic materials. This proof-of-concept research was inspired by the most recent review of functionalized aerogels [45], and it was assumed that the chelating N-donor centers in the aerogel [56,57,58,59,60] would facilitate the covalent binding of cobalt(II) ions in terms of manifesting slow magnetic relaxation behavior, similar to crystalline SIMs [61]. To immobilize the cobalt ions, the silica framework was chemically modified with chelating ethylenediamine moieties. Due to the diversity of the sol-gel approach, the concentration of ligands on the surface of a porous silica matrix can easily be tuned to attain a low, or high, concentration of paramagnetic centers in the material and to vary the energy barrier of magnetization, which is a key SIM characteristic, from 4 to 18 K. This approach opens up new possibilities for the design of advanced functional materials, with the synergy of an open porous structure and diluted magnetism.

## 2. Results and Discussion

### 2.1. Immobilization of Cobalt(II) Ions on Silica

For the chemical immobilization of Co^2+^ ions in the silica gel matrix, the co-gelation of methyltrimethoxysilane (MTMS) and N-[3-(trimethoxysilyl)propyl]ethylenediamine (EDTMS) was used, where amino groups of EDTMS were expected to strongly bind cobalt ions [62] during their extraction from the cobalt-containing solutions [63]. To synthesize silica aerogels bearing ethylenediamine moieties, tetra alkoxysilanes are commonly used [58,64,65,66]. The materials obtained by the co-gelation of EDTMS and tetraethoxysilane (TEOS) or tetramethoxysilane (TMOS) have very low mechanical strength; they are very brittle and awkward to handle. MTMS-based aerogels are known to be relatively strong, and even elastic; they have relatively high hydrophobicity [67], which is an advantage for the use of aerogel-based materials in moisture-containing environments. Surprisingly, no reports on the synthesis of ethylenediamine-modified silica aerogels by the co-gelation of MTMS and EDTMS were found. Experiments conducted during the current research showed that these two alkoxysilanes can easily be co-gelated to obtain easy-to-handle monoliths. Moreover, the formation of a silica network through the hydrolysis of MTMS leads to fewer Si–O and Si–OH bonds that are also capable of cobalt bonding [68,69]. According to the authors’ observations and previously published reports, the rate of MTMS hydrolysis in the presence of EDTMS is much slower than that of TMOS or TEOS; therefore, silica lyogels can be obtained in a more controlled manner [70,71]. Thus, the choice of MTMS favors the synthesis of silica aerogels, where the cobalt content can easily be controlled by the concentration of EDTMS in the gelling mixture.

The increase in EDTMS content in the reaction mixtures from 0 to 15 mol.% led to a drastic decrease in gelation time, from 2 days (for the pure MTMS) to 5 min, which was obviously due to the highly basic nature of the diamine moiety, which promoted silicon alkoxide polycondensation reactions as a result of the increase in pH [72]. The colorless wet-gel monoliths thus obtained were soaked in isopropanol solutions of cobalt(II) nitrate. Several minutes after being dipped in the solutions, the wet gels started to change their color to violet, due to the adsorption of cobalt(II) ions. According to the visual observations made while blade-cutting the test specimens, after 24 h, the whole volume of the wet gels was colored uniformly.

**Figure 1:**
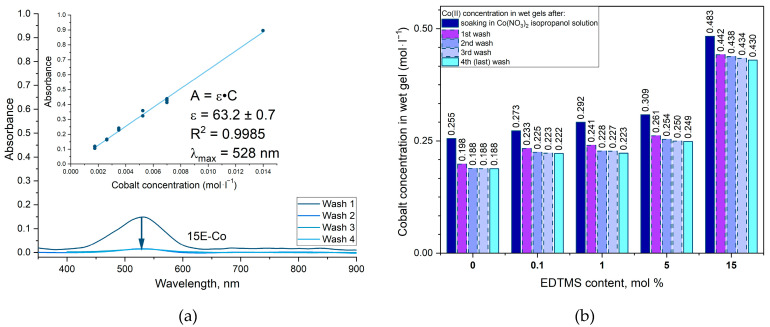
(**a**) Cobalt content in **15E-Co** wet gel after washing with isopropanol, showing the UV-vis absorbance spectra of washing liquors. Inset: UV-vis absorbance calibration curve of cobalt(II) nitrate isopropanol solutions. (**b**) Changes in cobalt(II) content in the wet gels after washing with isopropanol, as assessed using UV-vis spectroscopy of the initial solutions and the washing liquors.

After soaking, the wet gels were washed with isopropanol four times, to remove the cobalt ions that were weakly bound to the silica matrix. The cobalt(II) content in the washing liquors was monitored using UV-vis spectroscopy (Figure 1a). In the UV-vis spectra of the first washing liquors, an absorption peak at 528 nm was observed, corresponding to a ^4^*T*_1_ → ^2^*T*_1_ transition for a high-spin cobalt(II) ion [73] (Figure 1a, inset). Upon further washing, the intensity of the absorption decreased drastically, and the fourth washing liquor was colorless for all samples, indicating the complete removal of weakly bound cobalt ions from the wet gels.

The analysis of washing liquors enabled an estimation of the cobalt(II) content in the washed wet gels; they adsorbed 13–28% of the cobalt(II) from the soaking solutions (Figure 1b). The introduction of ethylenediamine moieties into the silica wet gels increased their cobalt sorption capacity by 90%. For the **0E-Co** sample prepared using 0% EDTMS, the Co(II) content in the gel was 0.255 M, while for the **15E-Co** sample containing 15% EDTMS, the Co(II) content reached 0.483 M. The most significant desorption of cobalt ions from the wet gels was observed for the first wash (8.5% to 22.4% of adsorbed cobalt ions). For the silica matrices with a higher content of ethylenediamine, the desorption of cobalt ions was much less, indicating the binding of the cobalt species to silica. Relative cobalt(II) desorption during the second wash reached 1–5% and further washes showed negligible (<2%) desorption for all the samples. After washing was complete, the wet gels retained 10–25% of the cobalt ions from the initial isopropanol solutions. Thus, the co-gelation of MTMS with various amounts of EDTMS enables the synthesis of wet gels that are capable of irreversibly adsorbing different amounts of cobalt ions. This approach encourages the synthesis of silica aerogels containing a controlled (high or low) concentration of immobilized metal ions.

**Figure 2:**
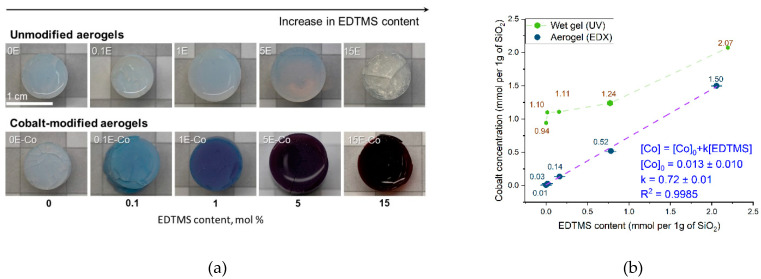
(**a**) Appearance of the prepared aerogels. (**b**) Control of cobalt concentrations by varying EDTMS content in the obtained wet gels (from UV-vis) and aerogels (from EDX).

The synthesized wet gels were dried in supercritical CO_2_ and cylindrical aerogel monoliths were obtained (Figure 2a) with ~40 vol.% of shrinkage. Cobalt-free silica aerogel (**E**) samples were opaque and slightly milky. In the aerogels, no visible cracks were observed, except in the case of the **15E** sample with maximum EDTMS content. Most probably, the low mechanical strength of the sample was due to a very high gelation rate, which resulted in the low chemical homogeneity of the monolith.

In turn, all the cobalt-containing aerogels (**E-Co**) were violet in color, with coloration increasing with the corresponding increase in the EDTMS content in the gels (Figure 2a).

The cobalt content of aerogels was measured using EDX analysis (Figure 2b). The supercritical drying of wet gels resulted in a drastic decrease in cobalt content. For example, cobalt content in the **1E-Co** sample decreased from 2.07 mmol·g^−1^ (SiO_2_) in the washed wet gel to 1.50 mmol·g^−1^ (SiO_2_) in the aerogel. Most probably, supercritical drying resulted in the removal of the cobalt ions bonded by Si–OH groups of the silica matrix. The relatively weak bonding of cobalt to silanol groups was expected, bearing in mind the high lability of [Co(OH)_n_] moieties [68,69]. During supercritical drying, 23–99% of the cobalt ions were desorbed from the wet gels. Higher desorption values were observed for those gels with a lower ethylenediamine content. This observation confirms the superior cobalt(II) binding by ethylenediamine-modified silica, compared with bare SiO_2_.

The desorption of cobalt(II) from the wet gels in supercritical CO_2_ was confirmed in a control experiment. The samples of **15E** and **15E-Co** wet gels were put together in an autoclave and supercritically dried. After this, the colorless **15E** sample was stained violet.

In the **0E-Co** aerogel, cobalt(II) concentration was very low, at 0.01 mmol·g^−1^ (SiO_2_), indicating weak cobalt(II) retention by silica bearing no ethylenediamine groups. In turn, for the aerogels containing ethylenediamine groups, a linear correlation (*R*^2^ = 0.9985) was observed between EDTMS content and cobalt(II) concentration. Such a linear correlation enables the assumption that, in the aerogels, all cobalt(II) ions are coordinated with the ethylenediamine ligands immobilized in the silica matrix. The linear regression coefficient (*k*) was equal to 0.72 ± 0.01 (Figure 2b), indicating that each cobalt(II) ion was presumably chelated with no more than a single ethylenediamine ligand.

**Figure 3:**
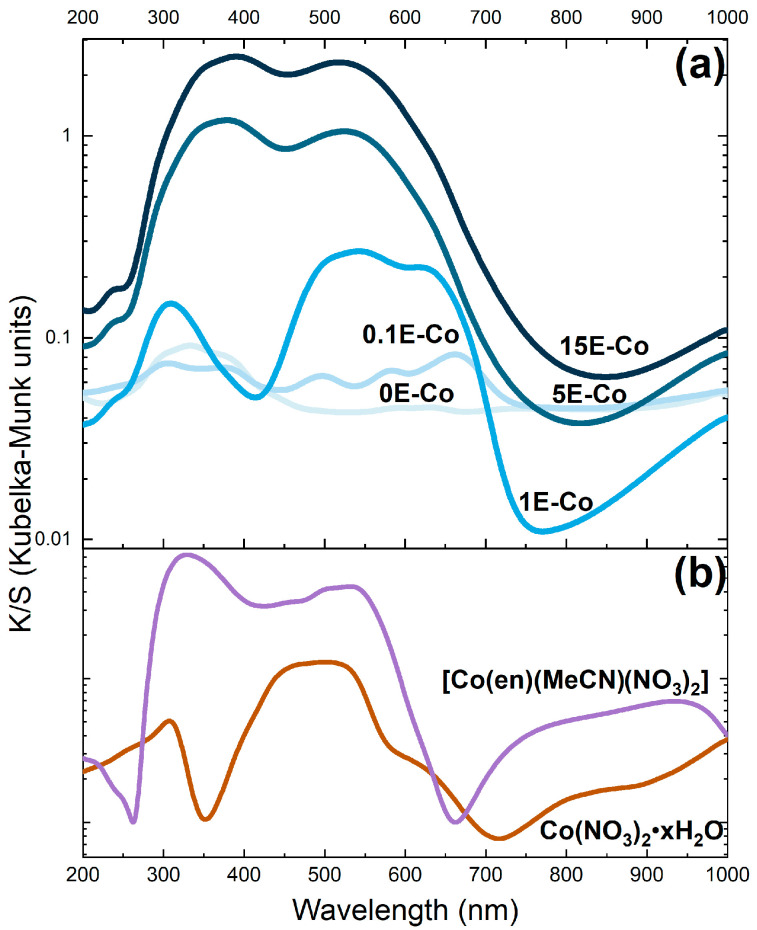
Diffuse reflectance spectra of (**a**) cobalt-modified aerogels, (**b**) Co(NO_3_)_2_·*x*H_2_O, and [(Co(en)(MeCN)](NO_3_)_2_].

To understand the chemical nature of cobalt complexes in silica aerogels bearing ethylenediamine groups, the samples were analyzed using UV-vis diffuse reflectance spectroscopy (Figure 3). The absorption of the samples containing no cobalt ions (**E** series) was negligible (Figure S1 in the Supplementary Materials); the absorption values were < 0.03 in Kubelka–Munk units.

For **0E-Co**, broad low-intensity bands with maxima at ~600 nm and above 1000 nm (the band maximum lay outside the spectral region of the device used, see Figure 3) can be attributed to *d*-*d* transitions for high-spin cobalt(II) in an octahedral environment of weak ligands, similar to [Co(H_2_O)_6_]^2+^ [74,75,76,77]. The spectra of **0.1E-Co** contain three new bands at 496, 582, and 662 nm, which may be due to the coordination of NH-groups with cobalt ions; similar spectral changes were observed during NH_3_ sorption by zeolites doped with cobalt(II) ions [78]. An increase in the concentration of ethylenediamine, as well as that of cobalt(II) (samples **1E-Co**, **5E-Co**, **15E-Co**), was accompanied by an increase in the intensity of the bands in the region of 450–750 nm and the wide band in the infrared region (>1000 nm).

The positions of the spectral maxima and their shape differed drastically from the cobalt(II) nitrate powder that was used for the preparation of aerogels (Figure 3). The difference in the intensity of the bands in the spectra of the **0E-Co** and **0.1E-Co** samples in the range of 450–750 nm indicates differences in the coordination environment of cobalt ions, presumably because of the transition from CoO_6_ to CoN_2_O_4_.

**Scheme 1:**
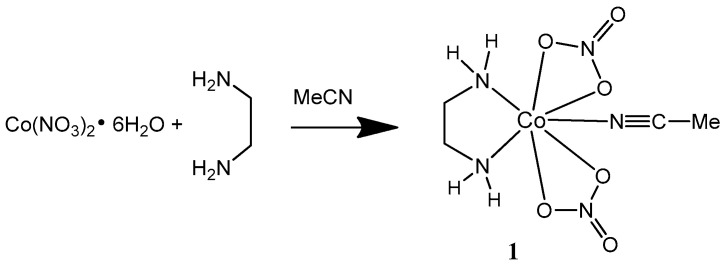
Synthesis of complex **1**.

To analyze the possible colorations of cobalt(II) ethylenediamine complexes, and to design a model compound with a cobalt(II) coordination similar to that in the aerogel matrix (ratio of Co:en = 1:1), a new [Co(en)(MeCN)(NO_3_)_2_] complex was synthesized (see Scheme 1). Note that, upon the exposure of the reaction mixture to atmospheric oxygen, yellow crystals of the previously reported cobalt(III) complex, [Co(en)_3_]·(NO_3_)_3_, were formed [78]; therefore, ethylenediamine complexes of divalent cobalt can only be obtained in an inert atmosphere [79,80,81,82,83].

**Figure 4:**
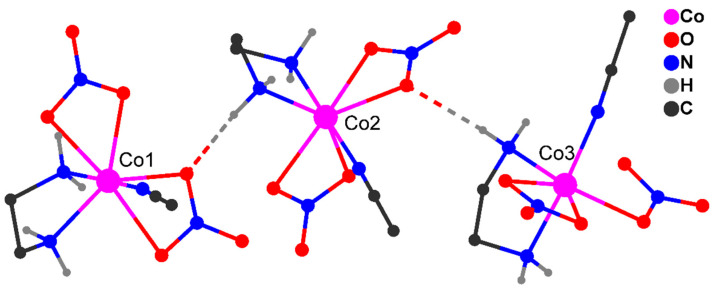
Molecular packing fragment of **1** (H atoms at carbon atoms are not shown; H-bonds are indicated by dashed lines).

The molecular structure of **1** was determined according to the single-crystal X-ray data; it crystallizes in the triclinic system in the space group *P*–1 as three independent molecules of the same composition (Figure 4). The molecules comprising Co1, Co2, and Co3 atoms have a similar structure, with each metal atom coordinating one bidentate ethylenediamine molecule (Co1–N 2.095(7), 2.113(6) Å; Co2–N 2.107(6), 2.116(6) Å; Co3–N 2.099(7), 2.108(7) Å), two bidentate nitrate anions (Co1–O 2.183(6)–2.376(6) Å; Co2–O 2.191(6)–2.365(6) Å; Co3–O 2.177(6)–2.505(6) Å), and a monodentate acetonitrile molecule (Co1–N 2.105(6) Å; Co2-N 2.066(7) Å; Co3-N 2.094(6) Å). The coordination environment of cobalt atoms (CoN_3_O_4_) corresponds to a pentagonal bipyramid (*S*_Q_(Co1) = 2.080, *S*_Q_(Co2) = 1.923, *S*_Q_(Co3) = 2.458), where the O atoms and one of the N atoms of the ethylenediamine molecule are in the equatorial plane. In the molecule comprising the Co3 atom, the CoN_3_O_4_ polyhedron is distorted at the highest extent compared with other independent molecules in the crystal cell, which is due to the increased Co3–O distance (2.505(6) Å). Formally, the coordination environment of the Co3 atom can be considered as 6 + 1. In the crystal, the molecules of the complex are linked by H-bonds to form a three-dimensional structure (Table S1 in the Supplementary Materials). The Cambridge Structural Database contains 122 cobalt compounds with a CoN_3_O*_x_* (*x* = 3, 4) coordination environment similar to that observed in **1**.

The coordination number (CN) of cobalt(II) atoms in the coordination compounds can be from 4 to 8. The compounds with CN = 7 are formed with small chelate ligands (e.g., nitrates, polychelate N,O-donors, etc.) [84,85,86,87]. Among the cobalt(II) nitrate complexes, compounds bearing either two or three ethylenediamine chelating ligands have been reported [88,89]; thus, complex **1** is the first example of a cobalt(II) nitrate complex with one ethylenediamine ligand in the coordination sphere of the metal atom.

The UV-vis spectrum of **1** shows bands at ~450 nm and ~520 nm, with shoulders at ~650 nm, ~850 nm, and ~950 nm, which presumably correspond to the cobalt(II) ion in the octahedral and pentagonal bipyramid environment (Figure 3). The comparison of the diffuse reflectance spectra of **1** and **XE-Co** enables the assumption that, upon the binding of cobalt(II) to the chelating group of hydrolyzed N-3-(trimethoxysilyl)propyl ethylenediamine, similar fragments with CN = 7 are formed, while, upon supercritical drying, the removal of volatile solvent molecules results in the stabilization of cobalt complexes with CN = 6. The additional stabilization of the fragments {Co(H_2_N-(CH_2_)_2_-NH-R)O_4_} in the aerogel structure is assumed to occur due to the H-bonding of the coordinated NH groups and oxygen atoms of the matrix, similar to that of **1**.

The above observations confirm the chemical immobilization of cobalt(II) ions in the silica matrix due to the formation of an ethylenediamine complex in the octahedral coordination environment.

### 2.2. The Chemical Composition of the Silica Aerogels

To enable a detailed investigation of the chemical composition of the synthesized aerogels (both the cobalt-modified and bare SiO_2_ samples), FTIR and Raman spectroscopy studies were performed. The data obtained indicate the possible instability of the ethylenediamine moiety in silica aerogels synthesized using EDTMS.

**Figure 5:**
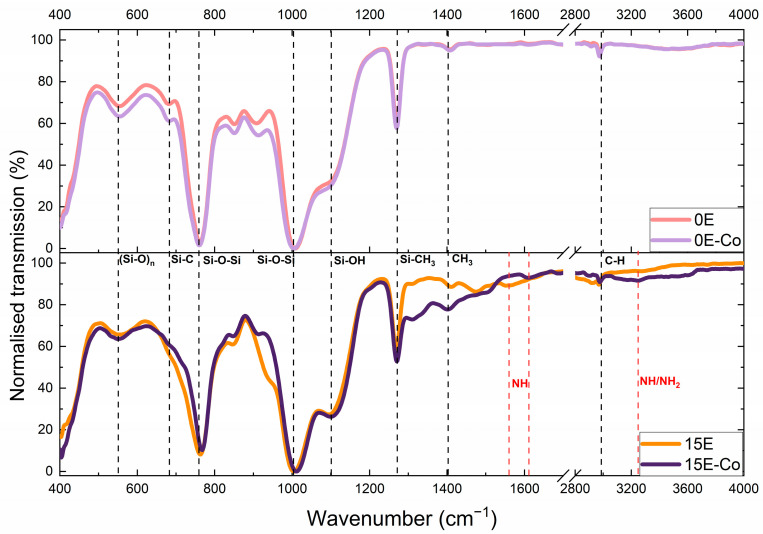
FTIR spectra of the cobalt-modified aerogels and the reference cobalt-free aerogels (0 mol.% and 15 mol.% of EDTMS).

For the cobalt-free aerogels, FTIR spectroscopy (Figure 5) confirmed the formation of a silica network. In the spectra of all the samples, absorption bands were observed that were attributed to the Si–O–Si (762 cm^−1^, 1008 cm^−1^) and Si–OH (1094 cm^−1^) bond vibrations [90] and the breathing (Si–O)_n_ modes (546 cm^−1^) [91]. In the aerogels, the presence of Si–CH_3_ fragments was indicated by absorption bands at 762 cm^−1^ (Si–C), 1270 cm^−1^ (Si–CH_3_), 1408 cm^−1^ (CH_3_), and 2974 cm^−1^ (C–H) [67,90]. The broad bands at 3200–3800 cm^−1^ were attributed to OH bond vibrations in the water and alcohol molecules adsorbed on the surface of the aerogels. The differences in the FTIR spectra of the aerogels synthesized using EDTMS and the aerogel synthesized from pure MTMS are almost negligible. In the FTIR spectrum of the **15E** aerogel, several extra-weak absorption bands are present in the range of 1400–1700 cm^−1^, and the band at 1552 cm^−1^ can definitely be attributed to the N–H bond vibrations [62]. It should be borne in mind that, due to the low polarity of the ethylenediamine moieties, FTIR spectroscopy can provide little information on the composition of ethylenediamine-modified aerogels.

**Figure 6:**
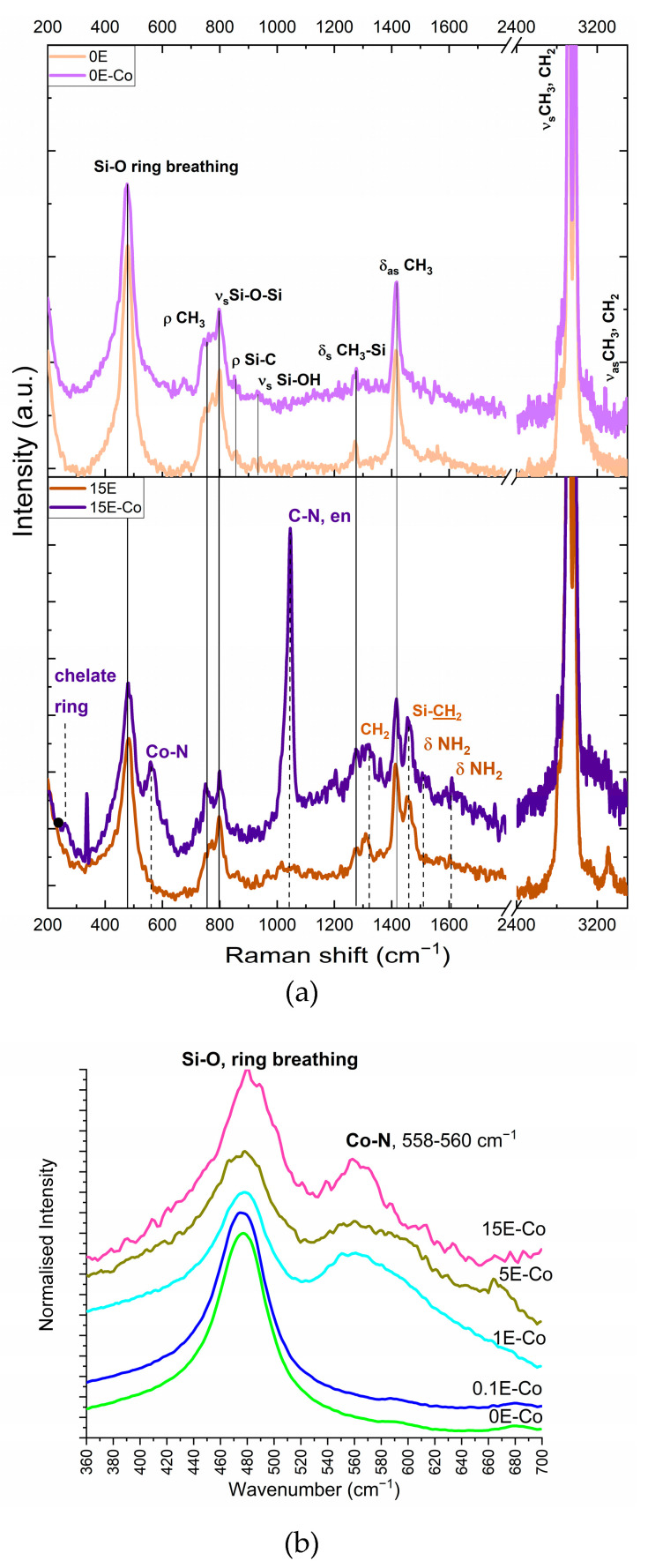
(**a**) Raman spectra of the aerogels; (**b**) Co–N bond vibration bands in the Raman spectra of the aerogels.

Generally, Raman spectroscopy is less sensitive to polar moieties but more sensitive to polarizable moieties than FTIR spectroscopy [92]. Thus, combining FTIR and Raman data makes it possible to obtain valuable information on the chemical composition of functionalized aerogels. In the Raman spectra (Figure 6) of the aerogels, the bands at 478 cm^−1^ ((Si–O)_n_ ring breathing), 800 cm^−1^ (ν_s_(Si–O–Si)), and 930 cm^−1^ (ν_s_(Si–OH)) indicate the formation of a SiO_2_ network. Evidence of the direct bonding of methyl groups to silicon atoms is provided by the bands at 1271 cm^−1^ (δ(Si–CH_3_)), 851 cm^−1^ (ρ(Si–C)), and 744 cm^−1^ (ρ(CH_3_)) [93]. Strong bands corresponding to the valence CH_2_ and CH_3_ vibrations from Si–CH_3_ groups and alcohol molecules are located in the 2800–3200 cm^−1^ range.

In the cobalt-free aerogels synthesized by the co-gelation of MTMS and EDTMS, the same bands are present. The most intriguing feature of the Raman spectra of these aerogels is the absence of any bands at ~1100 cm^−1^, where the C–N vibration of the ethylenediamine ligand was confidently expected [94,95]. The bands in the aerogels resemble those in the spectrum of (3-aminopropyl)-trimethoxysilane containing a Si–(CH_2_)_3_–NH_2_ fragment, at 1308 cm^−1^ (CH_2_), 1454 cm^−1^ (Si–CH_2_−), and 1603 cm^−1^ (NH_2_) [96]. This unexpected result indicates the absence of the ethylenediamine moiety in the aerogels prepared by co-gelation of MTMS and EDTMS. The decomposition of the Si–(CH_2_)_3_–NH–(CH_2_)–NH_2_ fragment is almost impossible during the sol-gel transition, and subsequent washing most likely happened during the supercritical drying stage. Some recent reports have discussed the possibility of ethylenediamine decomposition in the presence of water and carbon dioxide molecules, through the formation of intermediate carbamates [97,98,99]. Thus, decomposition reactions in ethylenediamine-modified silica gels are presumably favored by high CO_2_ pressures.

Importantly, Raman spectroscopy is rarely used for the analysis of ethylenediamine-modified silicas (see, e.g., [42,100,101,102]). The results of the current study indicate that the use of this method is very much needed.

The disappointing result described above became almost impressive when the Raman spectra of the cobalt-containing ethylenediamine-modified silica were analyzed. Here, the strong C–N bond vibration bands could be clearly observed at 1096 cm^−1^, indicating that cobalt ions encouraged the stability of the ethylenediamine moieties, most probably due to a strong chelating effect. Chelate ethylenediamine ring-bending vibrations can be observed at 260 cm^−1^; the band at 1515 cm^−1^ can be attributed to the NH_2_-bending vibrations in metal complexes [95]. In turn, the band at 558 cm^−1^ originates from Co–N bond vibrations [103]. In the Raman spectra of the **1E**, **5E**, and **15E** samples, the Co-N bond vibration bands can be observed at the same frequencies (558–560 cm^−1^, Figure 6b), confirming the similar coordination of cobalt atoms in all these samples [95].

Cobalt complex formation was also confirmed by the FTIR spectroscopy data (Figure 5). For example, the modification of aerogels with cobalt resulted in the shifting of the NH_2_ vibration band from 1552 cm^−1^ (**15E** aerogel sample) to 1606 cm^−1^ (**15E-Co** aerogel sample). Such a shift indicates cobalt ethylenediamine complex formation [62]. In the FTIR spectra, a weak band at 3232 cm^−1^ (**15E-Co** aerogel sample), can be attributed to NH-bond vibration [104].

Thus, Raman spectroscopy, a key method for analyzing ethylenediamine-modified silica, confirmed the presence of cobalt(II) complexes in the aerogels. In the complexes, amino groups are strongly coordinated with cobalt ions and their reduced basicity makes them less prone to chemical interaction with electrophilic CO_2_ molecules at high pressures. In the absence of metal ions, the ethylenediamine moiety presumably decomposes to yield primary amines. The exact mechanism of such a decomposition requires further comprehensive studies.

### 2.3. The Structural and Textural Properties of the Aerogels

As expected, the XRD patterns (Figure S2 in the Supplementary Materials) of the aerogel samples showed their amorphous structure, with no traces of any crystalline compounds. An amorphous structure is characteristic of silica aerogels containing chemically immobilized metal ions [105] or coordination compounds [42,106]. The Raman spectra (Figure 6) indicated no traces of cobalt oxides (e.g., Co_3_O_4_ with the characteristic Raman band at 694 cm^−1^) that could form upon the contact of aerogels with an ambient atmosphere [107].

**Figure 7:**
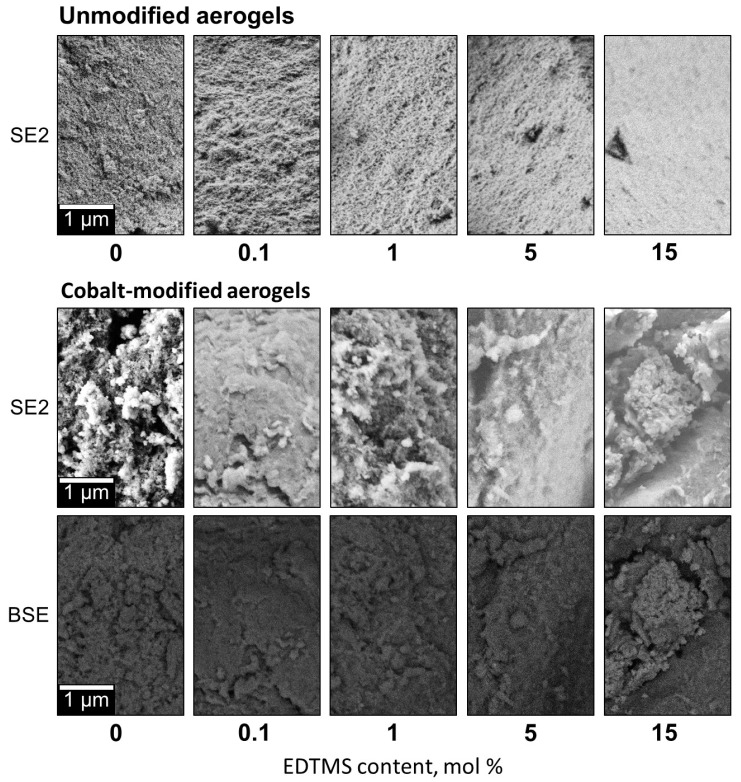
SEM images of the cobalt-modified and bare silica aerogels prepared using various amounts of EDTMS. SE2 and BSE represent the images registered at 1 kV in secondary electrons and back-scattered electrons, respectively.

All aerogels synthesized by the co-gelation of MTMS and EDTMS possessed a highly porous structure that was typical of silica aerogels [41], and evidence of this is provided by SEM images (Figure 7). The surface of the silica aerogel that was synthesized using the highest EDTMS concentration (**15E** sample) was quite smooth and contained virtually no mesopores. The surface of cobalt-modified aerogels is quite different from the bare silica samples, containing irregularly shaped aggregates and microcracks. However, back-scattered electron (BSE) images show no inhomogeneities in the cobalt’s spatial distribution over the aerogels’ surface; this was corroborated by the EDX mapping data (Figure S3 in the Supplementary Materials).

**Figure 8:**
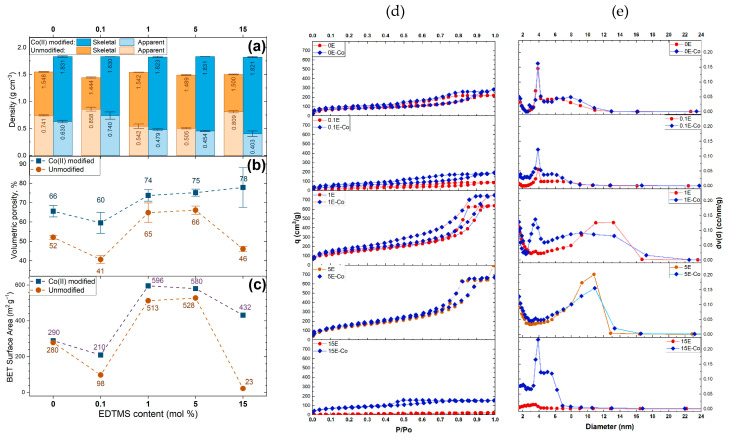
Textural properties of the cobalt-modified and bare aerogels: (**a**) skeletal and apparent density, (**b**) volumetric porosity, (**c**) BET surface area, (**d**) low-temperature nitrogen adsorption-desorption isotherms, (**e**) calculated BJH pore-size distribution for obtained aerogels (from the desorption branch).

Further investigations showed that modification of the gels with cobalt ions resulted in noticeable changes in their texture characteristics. The apparent density of all the aerogels, both bare and cobalt-modified aerogels, was in the range of 0.43–0.81 g·cm^−3^, with cobalt-modified aerogels having smaller apparent density values (0.43–0.75 g·cm^−3^) than cobalt-free aerogels (0.51–0.81 g·cm^−3^). The skeletal density of cobalt-modified aerogels was, in turn, significantly higher (1.82–1.83 g·cm^−3^) than that of bare silica (1.44–1.55 g·cm^−3^) (Figure 8a). These results show that the immobilization of cobalt in the gel structure resulted in an increase in the volumetric porosity of aerogels by 9–32% (Figure 8b). The exact reason for the higher volumetric porosity of cobalt-modified aerogels is unclear, but it might be hypothesized that it is connected to the higher chemical stability of the ethylenediamine moiety linked to cobalt ions under supercritical drying in CO_2_. Note that the opposite differences in volumetric porosity values were reported for ethylenediamine-modified silica aerogels containing the chemically immobilized heterobimetallic Zn-Cu complex [42].

Differences in the specific surface area values (*S*_BET_) for the aerogel samples are illustrated in Figure 8c. Cobalt-modified aerogels possessed higher specific surface area values than unmodified ones, except for the samples **0E** and **0E-Co**, which contained no ethylenediamine moieties. For these samples, the *S*_BET_ values were almost identical (280–290 m^2^·g^−1^). The highest *S*_BET_ values (500–600 m^2^·g^−1^) were registered for aerogels synthesized using 1–5 mol.% of EDTMS. The most striking difference in S_BET_ values can be observed for cobalt-modified and bare aerogels obtained using the highest EDTMS concentration (15 mol.%): 275 m^2^·g^−1^ for the **15E-Co** sample and 23 m^2^·g^−1^ for the **15E** sample. It is most probable that such a difference originates also from the higher chemical stability of the ethylenediamine moiety chelated to cobalt ions under supercritical drying conditions. Chemical decomposition reactions in the aerogels can result in pore shrinkage and the partial collapse of the porous structure, while the high stability of ethylenediamine linked to cobalt ions promotes the consistency of the aerogel network.

The differences in the porous structure of cobalt-modified and bare aerogels can readily be observed from low-temperature nitrogen adsorption–desorption isotherms (Figure 8d). Most of the full isotherms can be attributed to the IVb IUPAC type, which is inherent in the mesoporous structures and shows H2(b) hysteresis loops corresponding to pores with a wide distribution of neck widths [68]. Note that all the isotherms for the cobalt-modified aerogels showed higher adsorption at maximum nitrogen partial pressures, as well as wider hysteresis loops, corroborating the higher porosity of these samples compared with bare silica aerogels. The most significant information was obtained from the comparison of pore size distributions. Mesopores in all aerogels can be classified as being small (3–4 nm) or large (4–20 nm). As Figure 8e shows, the content of small mesopores was significantly higher for cobalt-modified aerogels than for bare aerogels synthesized using the same amount of EDTMS. Even in the **15E-Co** aerogel, small mesopores were present, while the **15E** aerogel showed a virtual absence of mesopores. This corroborates previous data indicating that modification with metal ions boosts the stability of the silica matrix, synthesized by the co-gelation of MTMS and EDTMS.

### 2.4. Magnetic Properties of Co-Modified Silica Aerogels

**Figure 9:**
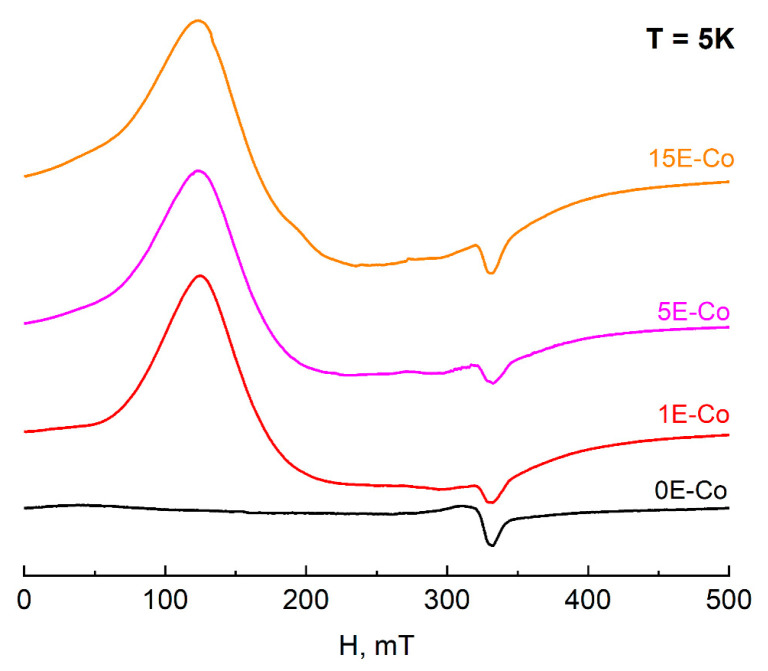
CW X-band EPR spectra of the aerogel samples **0E-Co**, **1E-Co**, **5E-Co,** and **15E-Co** at 5K.

At room temperature, all samples exhibited no electron paramagnetic resonance signals, which, in the case of high-spin Co^2+^ ions, could correspond to the magnetic relaxation being too fast. The cooling of the cobalt-containing samples to 5 K produced an intense EPR signal, with *g*-factor values of ~5.37–5.43 (Figure 9). High *g*-factor values (*g* > 2), along with the absence of an EPR signal at room temperature, are characteristic of high-spin Co^2+^ ions [108]. For all samples at *g* ~2 (*H* ~330 mT), a weak signal for the paramagnetic impurity present in the initial matrix can be observed.

**Figure 10:**
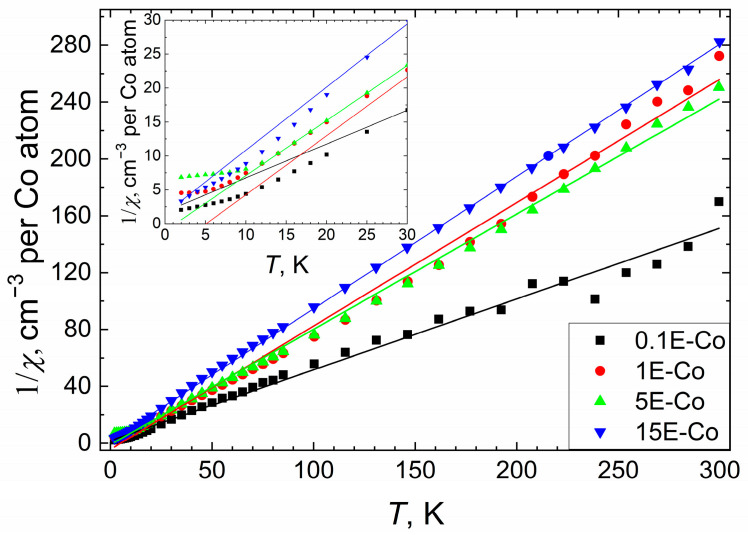
Temperature dependence of the inverse magnetic susceptibility as collected in an applied DC magnetic field of 0.5 T for aerogels with different EDTMS content (data calculated per Co atom, according to EDX results). Insert: low temperature range.

In order to determine the magnetic relaxation parameters of the materials obtained, detailed studies of the magnetic properties of the resulting aerogels were carried out. For all of the studied materials, magnetic susceptibility (χ = *M*/*H*) to temperature (*T*) dependencies had the form that was characteristic of paramagnets. To determine the presence of magnetic interactions in the samples, the dependencies of inverse magnetic susceptibility 1/*χ* on temperature were approximated (Figure 10) using the Curie–Weiss law equation, (*χ* = *C*/(*T* − θ), where *C* is the Curie constant, and *θ* is the Curie temperature. Best-fit parameters for all aerogels containing EDTMS are presented in Table S3 in the Supplementary Materials. The deviation of the Curie constant values from theoretical values for noninteracting cobalt ions Co^2+^ (*C* = 1.90 cm^3^·K·mol^−1^) can be associated with several factors, including: strong spin-orbit interaction; EDX method error in determining the real concentration of cobalt atoms in the samples; possible differences in the cobalt ion oxidation state in the sample from Co^2+^; the presence of strong antiferromagnetic interactions; the influence of the crystal field of the matrix. It should be noted that the dependencies 1/χ(*T*) deviate significantly from the linear behavior at temperatures below 20 K (insert on Figure 10), which may be associated with both the Zeeman effect (saturation) in an external magnetic field and the magnetic interactions between paramagnetic Co^2+^ ions.

To estimate the relaxation rate and determine the magnitude of the magnetization reversal barriers, AC-magnetic susceptibility was measured for all of the samples. In a zero magnetic field, slow magnetic relaxation (nonzero values of the imaginary component of the AC-magnetic susceptibility χ″) was observed for **1E-Co** only, but the maxima on the χ″ frequency dependence at 2 K was beyond the frequency range of the equipment used (Figure S4 in the Supplementary Materials). For lower EDTMS concentrations, the signal-to-noise ratio became unsatisfactory for the purposes of an unambiguous interpretation of the experimental data, even in nonzero magnetic fields. For higher EDTMS concentrations, slow magnetic relaxation in a zero magnetic field was not observed, most likely due to an increase in the relaxation rate as a result of the presence of magnetic (dipole–dipole) interactions between Co^2+^ ions.

The application of an external DC-magnetic field makes it possible to reduce the negative influence of the quantum tunneling effect, which can lead to an increase in the relaxation time of the system. In a DC-magnetic field, for all samples with an EDTMS content higher than 0.1%, the isotherms of the *χ*″ frequency dependences show non-zero values (Figures S5 and S6 in the Supplementary Materials), which confirms the presence of slow magnetic relaxation in the samples.

Measurements of the frequency dependencies of AC-magnetic susceptibility in optimal magnetic fields and their processing using the generalized Debye model (Figures S7–S9 in the Supplementary Materials) made it possible to determine the temperature dependences of the relaxation times of the samples with an EDTMS ligand content of 1%, 5%, and 15% (Figures S10–S12 in the Supplementary Materials). It should be noted that the deviation from the linear behavior of the dependences of relaxation time on the reciprocal temperature in semilogarithmic coordinates may indicate the influence of relaxation mechanisms other than the Orbach mechanism.

**Figure 11:**
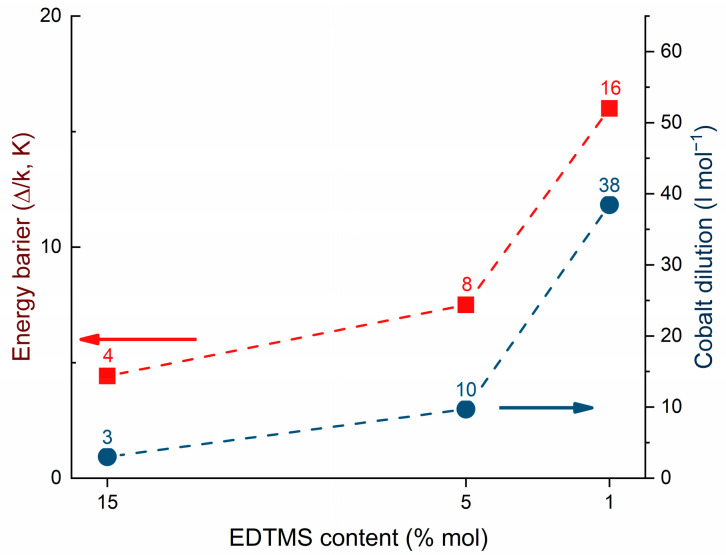
Magnetic dilution in aerogel-based cobalt SIM materials: cobalt atoms can be effectively dispersed in an aerogel matrix by lowering the EDTMS ligand content, thereby increasing the magnetic relaxation energy barrier.

The high-temperature section of the relationship between relaxation time and the temperature was approximated by the Arrhenius equation, *τ* = *τ*_0_ × exp{*Δ*/*kT*} (Orbach relaxation mechanism), in order to determine the effective value of the energy barrier of the magnetization reversal Δ of cobalt(II) ions in the matrices. From a best-fit analysis, the following values were obtained: *τ*_0_ = 6.6 × 10^−7^, 2.2 × 10^−6^ and 4.2 × 10^−6^ s^−1^; Δ/*k* = 18, 8 и 4 K, for **1E-Co**, **5E-Co** and **15E-Co**, respectively. An increase in the energy barrier of magnetization reversal with a decrease in EDTMS ligand content indicates the significant effect of magnetic (dipole–dipole) interactions between cobalt ions on magnetic relaxation in the samples (Figure 11).

Given that EPR spectra for **1E-Co**, **5E-Co** and **15E-Co** aerogels have the same shape, cobalt(II) ions in these materials have a similar electronic structure; on the other hand, the transition from **15E-Co** to **5E-Co** and **1E-Co** leads to an increase in the remagnetization of the energy barrier.

A similar effect of the concentration of paramagnetic centers on the energy barrier values was reported recently for cobalt(II) complexes entrapped in the diamagnetic crystalline matrices of their diamagnetic isostructural analogs [109]. For example, Colacio et al. reported on the Co(II)-Y(III) complex, [Co(L)(OAc)Y(NO_3_)_2_] (L is N,N′,N″-trimethyl-N,N″-bis(2-hydroxy-3-methoxy-5-methylbenzyl)-diethylenetriamine), which exhibited field-induced magnetic relaxation at 1000 Oe with Δ/*k* = 22.6 K [110]. The incorporation of [Co(L)(OAc)Y(NO_3_)_2_] in an isostructural Zn-Y analog (Co:Zn 1:10 mol.) resulted in an increase in the energy barrier to 27.1 K. SMM behavior was also reported for similar Co(II)-Y(III) benzoate or 9-anthracenecarboxylate complexes [111]. Similar results were obtained by Ceglarska et al. for Co_x_Zn_1–x_Br_2_(pyridine)_2_ complexes [112]. A decrease in cobalt(II) concentration from 91 to 6 at.% resulted in an energy barrier increase from 14 to 27 K. In metal–organic frameworks, the dilution of the cobalt(II) paramagnetic centers in a diamagnetic matrix was also reported as a possible strategy for the creation of SIM or SMM porous materials. A literature survey showed that, for SIM-MOFs, the energy barrier hardly ever exceeds 20 K [22,23,24,113]. The above results have demonstrated comparable concentration-affected changes in the magnetic behavior of the cobalt(II)-bearing silica aerogels obtained in the study, and of the recently reported cobalt(II)-containing materials.

## 3. Materials and Methods

### 3.1. Reagents

For the aerogel synthesis, methyltrimethoxysilane (MTMS, 98%, Sigma-Aldrich, St. Louis, MO, USA), N-[3-(trimethoxysilyl)propyl] ethylenediamine (EDTMS, 97%, Sigma-Aldrich, St. Louis, MO, USA), HCl (puriss. spec., Sigma-Tek, Moscow, Russia), NH_4_OH (25% water solution, puriss. spec., Khimmed, Moscow, Russia) and methanol (HPLC-grade, Khimmed, Moscow, Russia) were used. Co(NO_3_)_2_·6H_2_O (reagent grade, Khimmed, Moscow, Russia) was used to prepare isopropanol (reagent grade, Khimmed, Moscow, Russia) solution for wet gel soaking. Ethylenediamine dihydrochloride (98%, Reachem, Moscow, Russia), Co(NO_3_)_2_·6H_2_O (99%, Khimmed, Moscow, Russia), KOH (98%, Khimmed, Moscow, Russia), EtOH (96%, Khimmed, Moscow, Russia), and MeCN (99%, Khimmed, Moscow, Russia) were used for the synthesis of the [Co(en)(MeCN)(NO_3_)_2_] complex. All the experiments were conducted using deionized (18 MΩ) water.

### 3.2. Preparation of Cobalt-Modified Silica Aerogels

**Figure 12:**
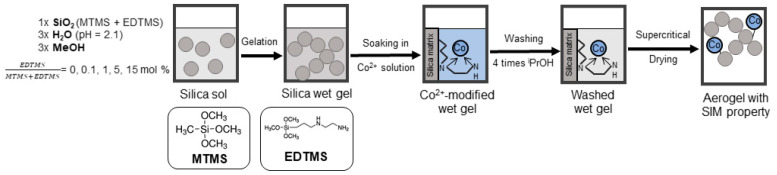
The preparation of silica-based cobalt-modified SIM materials.

The overall strategy of the synthesis is presented in Figure 12. It includes three main stages, which are described in subsequent subsections.

#### 3.2.1. Preparation of Ethylenediamine-Modified Silica Wet Gels

Silica wet-gel monoliths with various EDTMS contents, **X** (**X** = 0, 0.1, 1, 5, or 15 mol.%; $X=\frac{\nu \left(EDTMS\right)}{\nu \left(EDTMS\right)+\nu \left(MTMS\right)}\times 100\%$), were prepared for the further immobilization of paramagnetic cobalt ions. First, SiO_2_ sol was obtained by mixing $\frac{100-X}{100}$ molar parts of MTMS, 3 molar parts of methanol, and 3 molar parts of water, acidified to pH 2.1 with an aqueous HCl solution. After 30 min, the required volume of EDTMS was added to the SiO_2_ that was obtained. In all cases, the total volume of the reaction mixtures was 2.0 mL. The reaction mixtures were then immediately collected into 10 mL medical syringes. For the samples containing **X** = 1, 5, or 15 mol.% of EDTMS, gelation occurred within 1 h to 1 day, (the increase in EDTMS content accelerated gelation). For the samples containing **X** = 0 or 0.1 mol.% of EDTMS, gelation did not occur. For gelation of the latter samples, 100 μL of 2.5 vol.% aqueous ammonia was added to the reaction mixtures. To age the samples, all were stored in closed syringes for 3 days. Then, the syringes were opened and placed into 50 mL Corning Falcon^®^ tubes and washed with 35 mL of isopropanol. The resulting wet gels were either translucent (for **X** < 1 mol.%) or opaque (for **X** ≥ 1 mol.%).

#### 3.2.2. Modification of the Wet Gels with Cobalt Ions

The wet gels, prepared using various amounts of EDTMS, were soaked in 35 mL of isopropanol solution of cobalt nitrate ([Co^2+^] = 0.1049 M, as determined by ICP-OES) for 4 days. After the soaking stage, deep blue- or violet-colored wet gels were obtained. The soaked lyogels were washed four times with 35 mL of isopropanol, once a day. After one to three washes, the liquids were of a violet color; the coloration decreased from wash to wash. After the fourth washing, the liquids were colorless. The liquids used for soaking and washing were collected so that their cobalt concentration could be determined. The reference gel samples (containing no cobalt) were soaked in pure isopropanol.

#### 3.2.3. Supercritical Drying of Cobalt-Modified Lyogels

Drying in supercritical CO_2_ (*t*_cr_ = 31 °C, *P*_cr_ = 72.8 atm) was performed in an installation consisting of a Supercritical 24 high-pressure pump (SSI, Chicago, IL, USA), a 200 mL steel autoclave and a back-pressure regulator BPR (Waters, Milford, MA, USA). The samples were placed in an autoclave and submerged in isopropanol. The autoclave was closed and washed with supercritical CO_2_ at 50 °C, at 150 bar for 3 h. Within 30–40 min, the pressure was gradually decreased to atmospheric pressure and the autoclaves were cooled down to room temperature and opened. The aerogels that were prepared through soaking in cobalt(II) nitrate are labeled hereafter as “**X**E-Co”, where **X** corresponds to the molar content of EDTMS. The reference cobalt-free aerogel samples are labeled as “**X**E”.

### 3.3. Synthesis of [Co(en)(MeCN)(NO_3_)_2_]

Ethylenediamine dihydrochloride (0.1951 g, 0.002 mol) was added to a hot solution of potassium hydroxide (0.160 g, 0.004 mol) in ethanol (50 mL); the mixture was boiled under vigorous stirring for 20 min and the precipitate (sodium chloride) was filtered off. The filtrate was rigorously degassed and slowly added to an evacuated (5 × 10^−3^ bar) Schlenk tube, charged with a solution of Co(NO_3_)_2_·6H_2_O (0.2910 g, 0.001 mol) in ethanol (10 mL). The reaction mixture was heated up using a heat gun until boiling started, then left to cool at ambient temperature. A pink precipitate was formed in 5 min. Ethanol was removed by vacuum condensation and replaced with ca. 15 mL of acetonitrile. The resulting mixture was heated with a heat gun until boiling started, then left to cool at ambient temperature. Single crystals of the product that were suitable for X-ray diffraction studies had formed within 1 week. In an argon flow, the crystals were picked up directly from a Schlenk tube and immediately covered with oil. All attempts to obtain samples of [Co(en)(MeCN)(NO_3_)_2_] that were suitable for characterization by means of IR spectroscopy, elemental analysis, and powder X-ray diffraction failed, due to the extreme sensitivity of the compound to atmospheric oxygen and moisture. At the point of exposure of the complex to ambient conditions, a previously reported species [Co(en)_3_](NO_3_)_3_ [114] had formed.

### 3.4. Methods of Analysis

The cobalt concentration in the isopropanol solution used for the soaking of wet gels was determined by optical emission spectroscopy, with inductive-coupled plasma (ICP-OES), using a Thermo Scientific (Waltham, MA, USA) iCAP XP spectrometer. The reference-standard cobalt solution was used. The standard error in the three measurements did not exceed 2%.

UV-visible spectroscopy was performed using Ocean Optics (Orlando, FL, USA) equipment. The absorbance spectra of the washing liquors in Plastibrand PMMA cuvettes were recorded using a DH-2000 (20 W) deuterium–halogen light source with a wavelength of *λ* = 528 nm (absorption maximum); this was used for the determination of Co^2+^ concentration. Diffuse reflectance spectra (DRS) of aerogel samples were recorded using an HPX-2000 (35W) xenon light source and a 50-8-R-GT integrating sphere (Ø = 50 mm) against an Ocean Optics white standard. The signal collection time was 0.1 s for the absorbance spectra and 0.4 s for DRS measurements. All spectra were registered using an Ocean Optics QE 65,000 spectrometer.

The X-ray diffraction datasets for [Co(en)(MeCN)(NO_3_)_2_] crystals were collected using a Bruker (Billerica, MA, USA) D8 Venture diffractometer, equipped with a CCD detector (Mo_Kα_, *λ* = 0.71073 Å, graphite monochromator) [115]. Single crystal-diffraction pattern analysis revealed non-merohedral twinning. The orientation matrices for the two domains were determined using the Cell Now program [116]; both components were combined using Apex3. A semiempirical absorption correction was applied using Twinabs software [117]. Using Olex2 software [118], the structure was solved on the basis of unique domain 1 reflections with the ShelXT [119] and then refined using hkl 5 with the olex2.refine [118] refinement package, with least-squares minimization against *F*^2^ in an anisotropic approximation for non-hydrogen atoms. The hydrogen atoms of the ligands were positioned geometrically and then refined using the riding model. The crystallographic parameters for the [Co(en)(MeCN)(NO_3_)_2_] complex at *T* = 150(2) K were as follows: C_12_H_33_Co_3_N_15_O_18_, *fw* = 852.32, pink, parallelepiped, crystal size 0.30 x 0.20 x 0.10 mm, triclinic system, space group *P*-1, *a* = 8.995(3) Å, *b* = 12.664(4) Å, *c* = 15.648(5) Å, α = 107.867(9)°, *β* = 102.997(11)°, γ = 93.329(11)°, *V* = 1637.6(9) Å^3^, *Z* = 2, *ρ*_calc_ = 1.729 g·cm^−3^, *μ* = 1.596 cm^−1^, 9,682 measured reflections, 8,483 reflections with *I* > 2.0*σ*(*I*), *R*_int_ = 0.0388, *GooF* = 1.034, *R_1_* (*I* > 2*σ*(*I*)) = 0.0345, *wR_2_* (*I* > 2*σ*(*I*)) = 0.0421, *R_1_* (all data) = 0.0887, *wR_2_* (all data) = 0.0930, *T*_min/max_ = 0.563/0.746. CCDC 2212782 contains the supplementary crystallographic data for [Co(en)(MeCN)(NO_3_)_2_].

X-ray powder diffraction (XRD) patterns were recorded with a Bruker (Billerica, MA, USA) D8 Advance diffractometer, using Cu_Kα_-radiation (λ = 1.5406 Å) within a 2θ range of 5–90°, and with a signal collection time of 0.1 s per step.

Scanning electron microscopy (SEM) of the aerogels was performed on a Carl Zeiss (Oberkochen, Germany) NVision 40 high-resolution scanning electron microscope, equipped with an Oxford Instruments (Abingdon, UK) X-MAX (80 mm^2^) detector for energy-dispersive X-ray analysis (EDX). SEM images were recorded with an Everhart-Thornley (SE2) and back-scattered electron (BSE) detectors at 1 kV accelerating voltage, at a magnification of ×30,000. For the EDX analysis, a 20 kV accelerating voltage was used.

Fourier-transformed infra-red (FTIR) spectroscopy of the aerogels was performed using a PerkinElmer (Waltham, MA, USA) Spectrum 65 spectrometer equipped with an attenuated total reflectance (ATR) Specac Quest ATR accessory.

Raman spectra of the aerogels were recorded using a Renishaw InVia (Gloucestershire, UK) Reflex spectrometer, equipped with a 532 nm 30 mW laser using a ×50 magnification lens (N = 0.75) in the wavenumber range of 200–4000 cm^−1^, at 10% laser power.

The skeletal density ($\rho_{skel}$, g⋅cm^−3^) of the aerogels was measured with a Thermo Fisher Scientific (Waltham, MA, USA) Pycnomatic ATC helium pycnometer. The apparent density ($\rho_{app}$, g⋅cm^−3^) values were calculated for the cylindrical samples as $\rho_{app}=\frac{h\cdot \pi \cdot D^{2}}{4\cdot m}$, (here, h is the sample’s height, D—diameter, and *m*—weight). All measurements were performed five times and the precision of the height and diameter measurements was 0.02 mm; the precision of the weighing was 0.0001 g. Volumetric porosity (P, %) was calculated as $P=\left(1-\frac{\rho_{app}}{\rho_{skel}}\right)\times 100\%$.

Full low-temperature nitrogen adsorption–desorption isotherms were obtained using a QuantaChrome (Boynton Beach, FL, USA) Nova 4200B analyzer. Before the analysis, the samples were degassed at 100 °C in a vacuum for 16 h. Pore size distributions were calculated using a Barrett–Joyner–Halenda (BJH) model in the $P_{o}$ range of 0.01–0.99. From the isotherms obtained, the specific surface area *S*_BET_ for the samples was calculated using a Brunauer–Emmett–Teller (BET) model within the partial nitrogen pressure ($P_{o}$) range of 0.05–0.25. The applicability of the BET model was tested for each sample with respect to the criteria described elsewhere [120,121].

The electron paramagnetic resonance (EPR) experiments were run on a Bruker (Billerica, MA, USA) Elexsys E680 pulse/cw EPR spectrometer, equipped with an Oxford Instruments (Abingdon, UK) temperature control system (4–300 K). A standard Super High QE (SHQE) cavity resonator ER 4123SHQE (X-band) was used.

Magnetic behavior was studied using a Quantum Design (San Diego, CA, USA) PPMS-9 system. The temperature dependencies of the magnetization (*M*) were measured in a 0.5 T magnetic field in the temperature range of 2–300 K, during cooling. During AC susceptibility measurements in the frequency range of 10–10^5^ Hz, the alternating magnetic field amplitude was *H*_ac_ = 1–5 Oe. The measurements were carried out on samples that were moistened with mineral oil to prevent any texturizing of the particles in the DC magnetic field. The prepared samples were sealed into polyethylene bags. The magnetic susceptibility *χ* = *M*/*H* was determined, taking into account the contribution of the bag and that of the mineral oil. The data obtained were recalculated per cobalt atom, according to the EDX results.

## 4. Conclusions

The co-gelation of methyltrimethoxysilane with N-3-(trimethoxysilyl)propyl ethylenediamine resulted in ethylenediamine-modified silica wet gels that were capable of immobilizing cobalt(II) ions from isopropanol solutions. The variation of N-3-(trimethoxysilyl)propyl ethylenediamine content in the reaction mixtures made it possible to obtain wet gels with different concentrations of ethylenediamine moieties (0.1–15 mol.%) bonded to a silica matrix that governed chemisorption capacity. After low-temperature supercritical drying of the wet gels in CO_2_, cobalt-modified aerogels were produced that contained 0.01–1.50 mmol of Co(II) ions per 1 g of SiO_2_. In the absence of immobilized cobalt, free ethylenediamine moieties demonstrated low stability after supercritical drying and transformed into primary amines. Conversely, ethylenediamine moieties chelated to cobalt ions remained stable during the drying stage. Cobalt-modified aerogels were analyzed using different methods, including UV-vis spectroscopy, DRS, EDX, FTIR- and Raman spectroscopy and EPR, to confirm the successful chemical immobilization of Co(II) ions by an aerogel matrix bearing ethylenediamine moieties. To confirm the chemical bonding of Co(II) on the aerogel surface, a new cobalt(II) complex [Co(en)(NO_3_)_2_(MeCN)] was successfully synthesized and its structure was solved. Aerogels bearing Co(II) ions showed slow magnetic relaxation in a nonzero field, thus acting as diluted magnetic materials, representing the first examples of aerogel-based single-ion magnets.

## Data Availability

The structure parameters of obtained compound were deposited with the Cambridge Structural Database (CCDC No. 2212782; deposit@ccdc.cam.ac.uk or http://www.ccdc.cam.ac.uk/data_request/cif, accessed on 13 October 2022).

## Supplementary Materials

The following supporting information can be downloaded at: https://www.mdpi.com/article/10.3390/molecules28010418/s1, Figure S1: UV-VIS diffuse reflectance spectra of unmodified aerogels; Figure S2: XRD patterns of the aerogel samples; Figure S3: EDX mapping of the aerogel samples; Figure S4: AC frequency dependences of the real (χ ’, left) and imaginary (χ ’’, right) parts of AC susceptibility for **1E-Co** in different DC-magnetic fields and for AC frequencies between 10 Hz and 10,000 Hz at 2 K; Figure S5: AC frequency dependences of the real (χ ’, left) and imaginary (χ ’’, right) parts of AC susceptibility for **5E-Co** in different DC-magnetic fields and for AC frequencies between 10 Hz and 10,000 Hz at 2 K; Figure S6: AC frequency dependences of the real (χ’, left) and imaginary (χ’’, right) parts of AC susceptibility for **15E-Co** in different DC-magnetic fields and for AC frequencies between 10 Hz and 10,000 Hz at 2 K; Figure S7: AC frequency dependences of the real (χ’, left) and imaginary (χ’’, right) parts of AC susceptibility for **1E-Co** under a 2,500 Oe field at different temperatures; Figure S8: AC frequency dependences of the real (χ’, left) and imaginary (χ’’, right) parts of AC susceptibility for **5E-Co** under a 2,500 Oe field at different temperatures; Figure S9: AC frequency dependences of the real (χ ’, left) and imaginary (χ’’, right) parts of AC susceptibility for **15E-Co** under a 2,500 Oe field at different temperatures; Figure S10: Temperature dependence of the relaxation time for **1E-Co** estimated from the generalised Debye fits of the AC susceptibility data shown in Fig. S7 collected under a 2,500 Oe field; Figure S11: Temperature dependence of the relaxation time for **5E-Co** estimated from the generalised Debye fits of the AC susceptibility data shown in Figure S8 collected under a 2,500 Oe field; Figure S12: Temperature dependence of the relaxation time for **15E-Co** estimated from the generalised Debye fits of the AC susceptibility data shown in Figure S9 collected under a 2,500 Oe field; Table S1: Parameters of H-bonds in the crystal of [Co(en)(MeCN)(NO_3_)_2_]; Table S2: Texture properties of the aerogel samples; Table S3: Best-fit parameters of experimental magnetic data approximation using the Curie-Weiss equation (data calculated per Co atom according to EDX results).

## Sample Availability

Samples of the compounds are not available from the authors.
