# Supplementary material for: Aerogel-Based Single-Ion Magnets: A Case Study of a Cobalt(II) Complex Immobilized in Silica
Source: Molecules. 2023 Jan 3;28(1):418. doi: 10.3390/molecules28010418 (PMC9824035; doi:10.3390/molecules28010418)
Supplement: Supplementary file 1 [file molecules-28-00418-s001.zip › molecules-2055385-supplementary.docx]

Supplementary Materials:

Aerogel-Based Single-Ion Magnets: A Case Study of a Cobalt(II) Complex Immobilized in Silica

Sergey Yu. Kottsov, Maxim A. Shmelev, Alexander E. Baranchikov, Mikhail A. Kiskin, Alim U. Sharipov, Nikolay N. Efimov, Irina K. Rubtsova, Stanislav A. Nikolaevskii, Gennady P. Kopitsa, Tamara V. Khamova, Ilya V. Roslyakov, Igor L. Eremenko and Vladimir K. Ivanov

**Table S1.** Parameters of H-bonds in the crystal of [Co(en)(MeCN)(NO_3_)_2_].

| **Interaction** | Distance, Å | | | | Angle D–H…A, deg. |
| --- | --- | --- | --- | --- | --- |
|  | D–H | Symmetry code | H...A | D...A |  |
| **N4–H4A…O17** | 0.91 | 1+x,1+y,1+z | 2.43 | 3.158(3) | 137 |
| N4–H4B…O18 | 0.91 | 1+x,1+y,1+z | 2.39 | 3.096(7) | 131 |
| N5–H5A…O18 | 0.91 |  | 2.46 | 3.272(3) | 149 |
| N5–H5B…O10 | 0.91 | 1-x,1-y,2-z | 2.13 | 3.035(3) | 172 |
| N9–H9C…O16 | 0.91 | 1-x,-y,1-z | 2.31 | 3.310(3) | 171 |
| N9–H9D…O13 | 0.91 | 2-x,1-y,2-z | 2.37 | 3.153(3) | 144 |
| N10–H10С…O4 | 0.91 |  | 2.39 | 3.176(3) | 145 |
| N10–H10В…O2 | 0.91 | 1-x,1-y,2-z | 2.36 | 3.142(3) | 144 |
| N13–H13A…O11 | 0.91 |  | 2.20 | 3.055(3) | 156 |
| N13–H13B…O12 | 0.91 | 1-x,-y,1-z | 2.37 | 3.146(3) | 143 |
| N14–H13A…O5 | 0.91 | 1-x,1-y,1-z | 2.29 | 3.192(3) | 168 |
| N14–H13B…O9 | 0.91 | 1-x,1-y,1-z | 2.38 | 3.234(3) | 157 |
| C2–H2B…O3 | 0.98 | 2-x,1-y,2-z | 2.54 | 3.485(4) | 162 |
| C6–H6A…O15 | 0.98 | -x,1-y,1-z | 2.42 | 3.137(5) | 130 |
| C6–H6C…O9 | 0.98 | 1-x,1-y,1-z | 2.44 | 3.412(4) | 170 |
| C8–H8B…O2 | 0.98 |  | 2.50 | 3.273(3) | 134 |
| C12–H12A…O13 | 0.98 | -1+x,-1+y,-1+z | 2.52 | 3.369(4) | 145 |
| C12–H12B…O13 | 0.98 | -x,1-y,1-z | 2.50 | 3.289(4) | 138 |


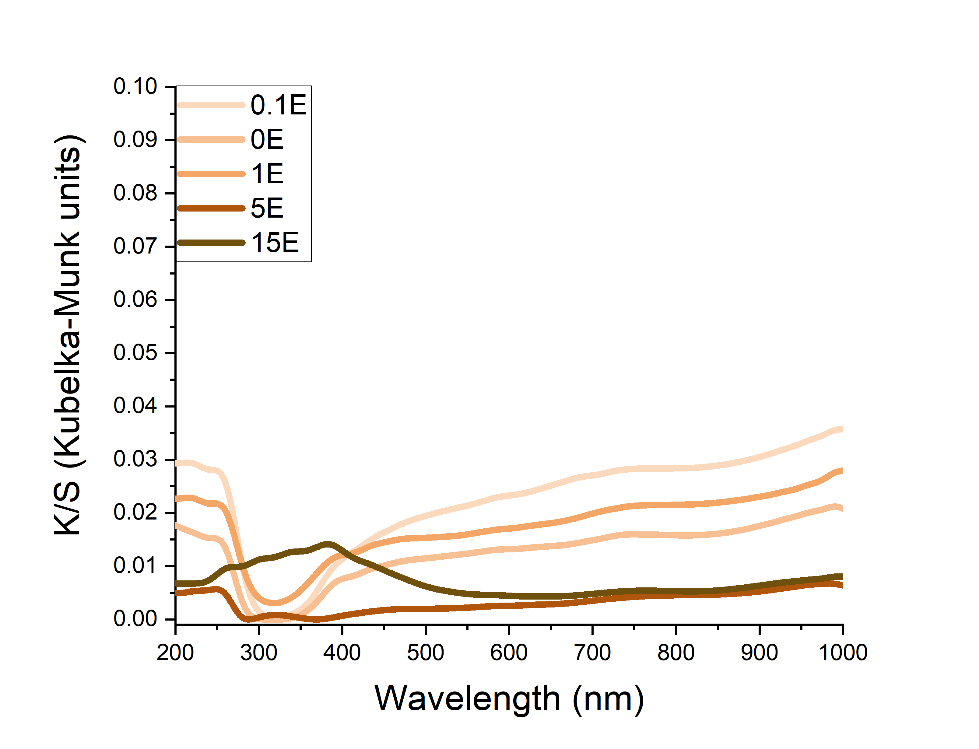


**Figure S1.** UV-VIS diffuse reflectance spectra of unmodified aerogels.


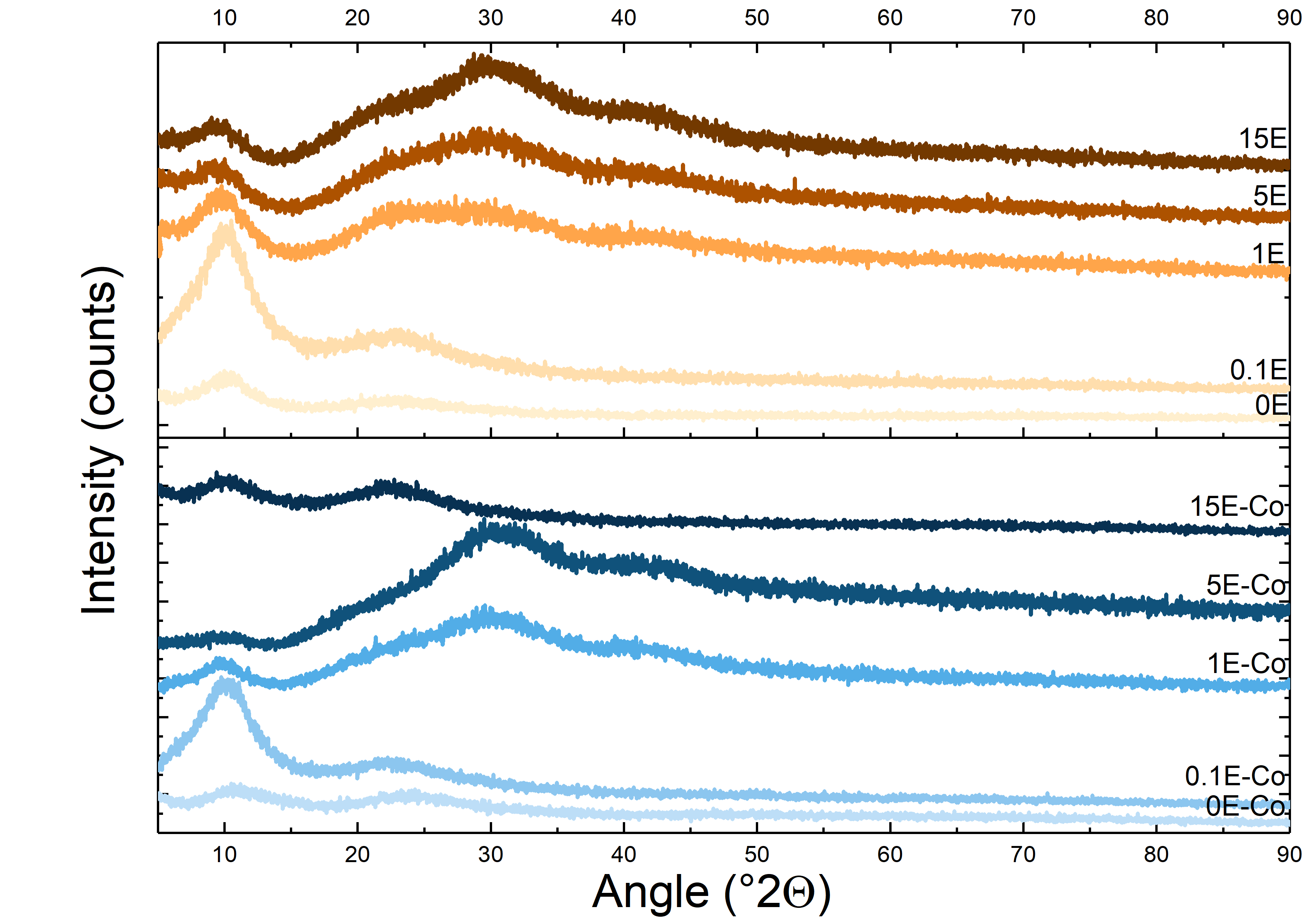


**Figure S2.** XRD patterns of the aerogel samples.


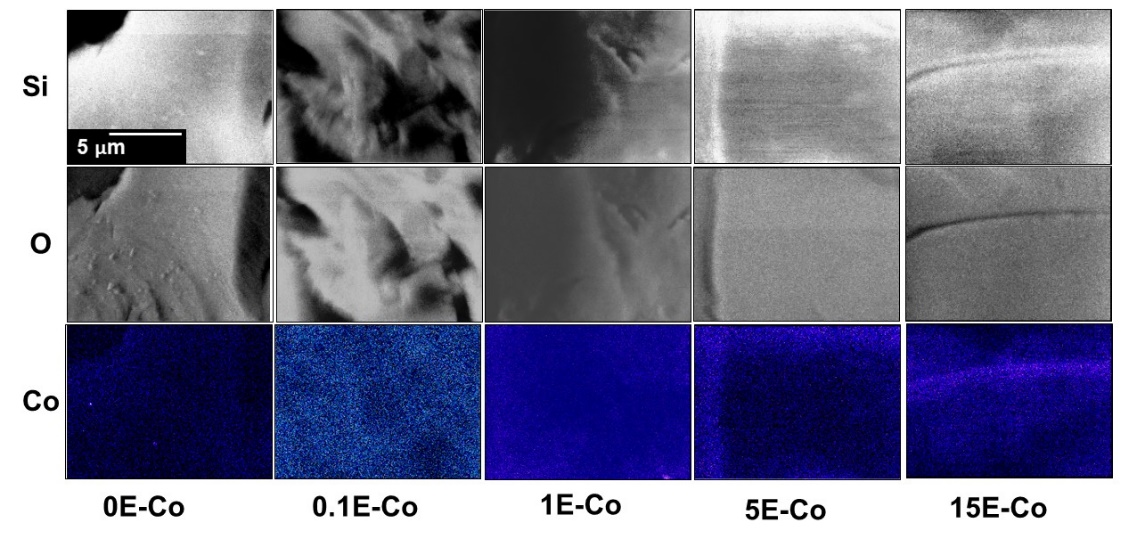


**Figure S3.** EDX mapping of the aerogel samples.

**Table S2.** Texture properties of the aerogel samples.

| **EDTMS content, %** | **0** | | **0.1** | | 1 | | 5 | | 15 | |
| --- | --- | --- | --- | --- | --- | --- | --- | --- | --- | --- |
| **Cobalt-modified?** | **No** | **Yes** | **No** | **Yes** | No | Yes | No | Yes | No | Yes |
| *S*_BET_, m^2^⋅g^–1^ | 280 | 289 | 98 | 210 | 513 | 596 | 528 | 580 | 22.9 | 432 |
| *C*_BET_ | 115 | 865 | 34 | 31 | 61 | 117 | 44 | 49 | 6 | 34 |
| BJH cumulative pore volume, cm^3^⋅g^–1^ |  |  |  |  |  |  |  |  |  |  |
| by adsorption | 0.32 | 0.39 | 0.15 | 0.31 | 1.00 | 1.13 | 1.27 | 1.07 | 0.04 | 0.22 |
| by desorption | 0.30 | 0.40 | 0.11 | 0.29 | 0.98 | 1.16 | 1.27 | 1.07 | 0.04 | 0.24 |
| BJH mean pore size, nm |  |  |  |  |  |  |  |  |  |  |
| by adsorption | 1.5 | 1.5 | 1.5 | 1.7 | 1.5 | 1.5 | 1.5 | 1.5 | 3.3 | 1.5 |
| by desorption | 3.9 | 3.9 | 3.9 | 3.9 | 13.2 | 3.6 | 10.8 | 10.9 | 3.6 | 3.5 |

**Table S3.** Best-fit parameters of experimental magnetic data approximation using the Curie-Weiss equation (data calculated per Co atom according to EDX results).

| **Sample** | ***C*, cm^3^⋅K/mol** | ***θ*, K** |
| --- | --- | --- |
| **0.1E-Co** | 2.00 (±0.04) | –3.6 (±2.5) |
| **1E-Co** | 1.15 (±0.01) | 5.1 (±1.5) |
| **5E-Co** | 1.23 (±0.01) | 1.2 (±1.0) |
| **15E-Co** | 1.07 (±0.01) | –1.6 (±0.4) |

| 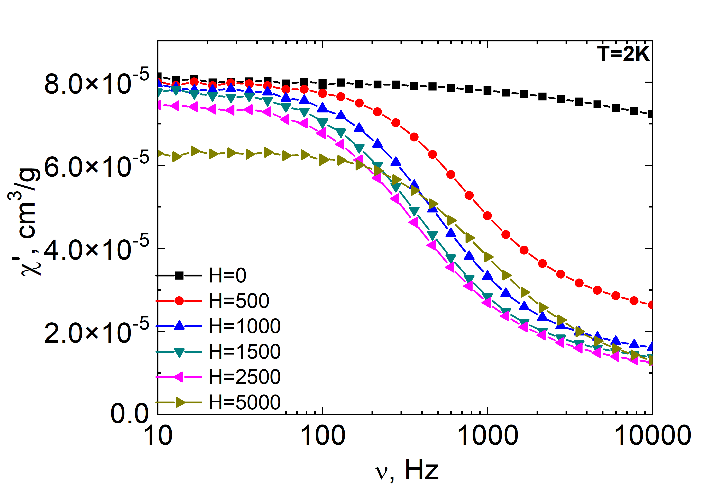 | 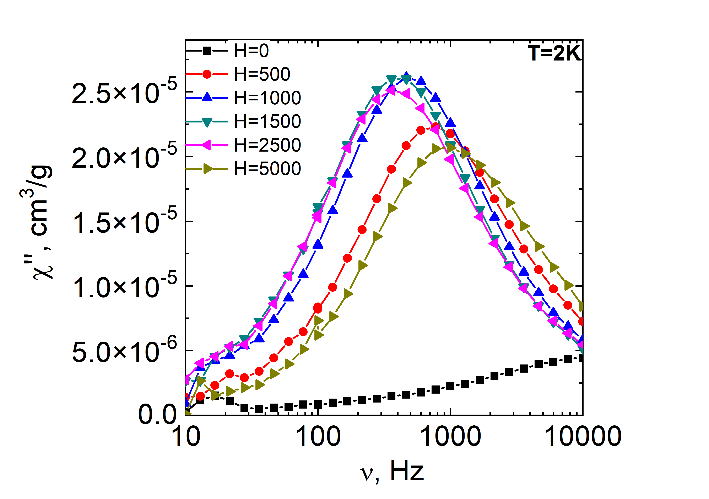 |
| --- | --- |

**Figure S4.** AC frequency dependences of the real (χ ', left) and imaginary (χ '', right) parts of AC susceptibility for **1E-Co** in different DC-magnetic fields and for AC frequencies between 10 Hz and 10,000 Hz at 2 K.

| 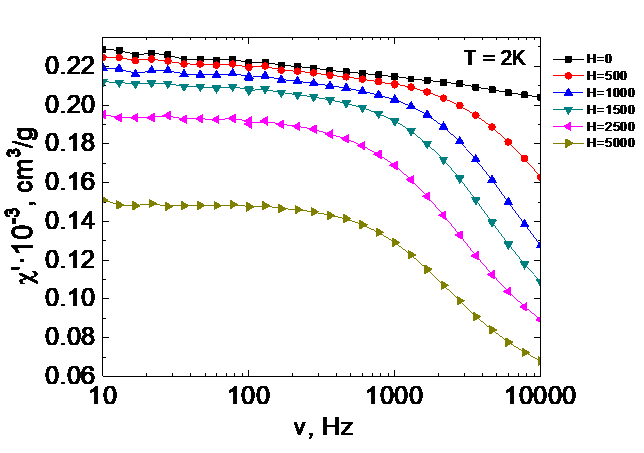 | 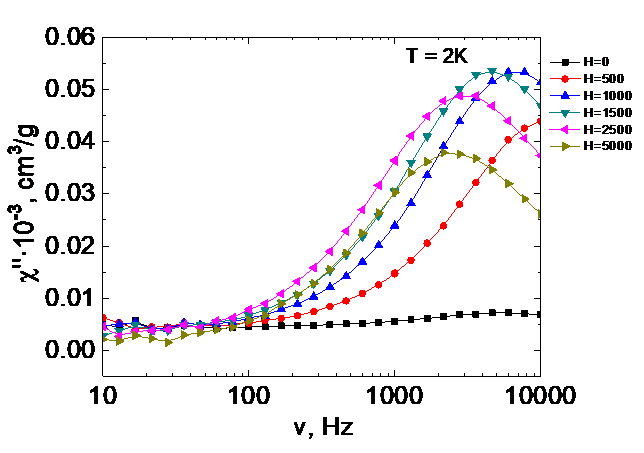 |
| --- | --- |

**Figure S5.** AC frequency dependences of the real (χ ', left) and imaginary (χ '', right) parts of AC susceptibility for **5E-Co** in different DC-magnetic fields and for AC frequencies between 10 Hz and 10,000 Hz at 2 K.

| 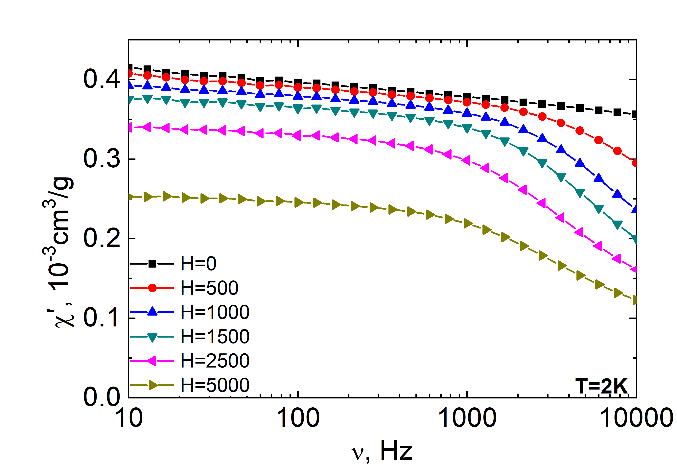 | 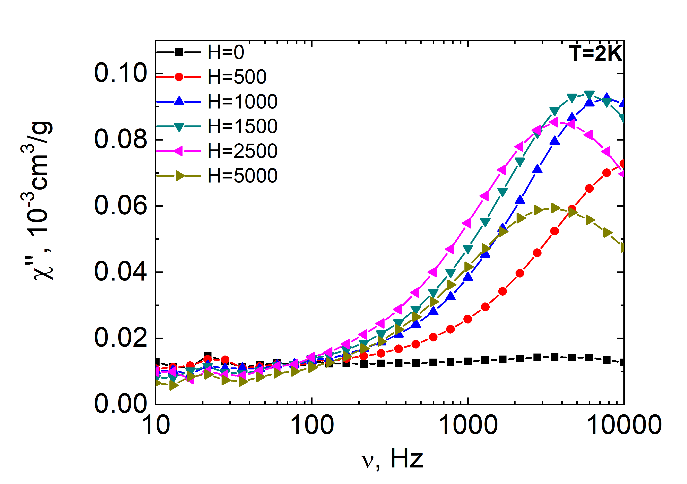 |
| --- | --- |

**Figure S6.** AC frequency dependences of the real (χ', left) and imaginary (χ'', right) parts of AC susceptibility for **15E-Co** in different DC-magnetic fields and for AC frequencies between 10 Hz and 10,000 Hz at 2 K.

| 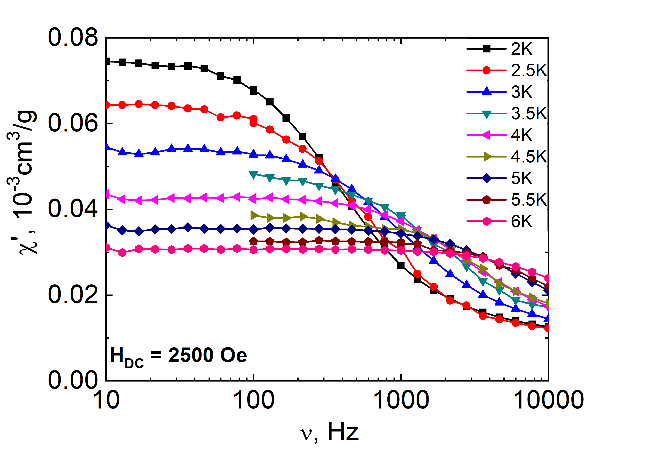 | 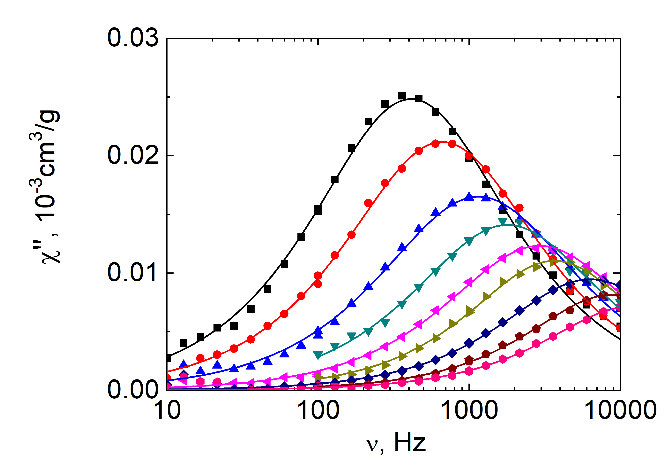 |
| --- | --- |

**Figure S7.** AC frequency dependences of the real (χ', left) and imaginary (χ'', right) parts of AC susceptibility for **1E-Co** under a 2,500 Oe field at different temperatures.

| 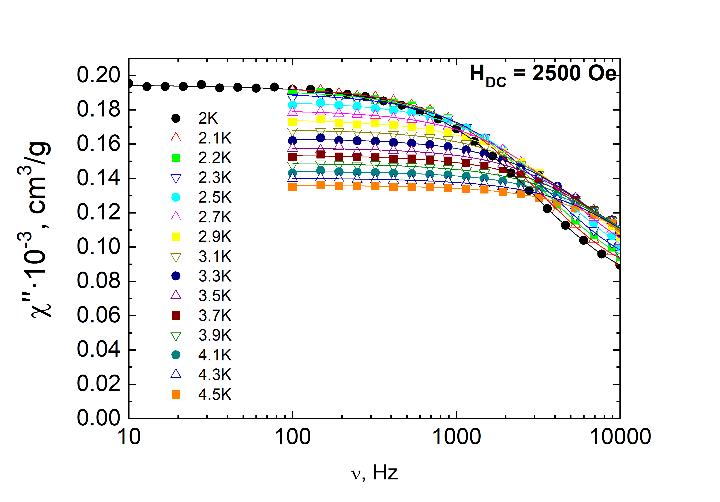 | 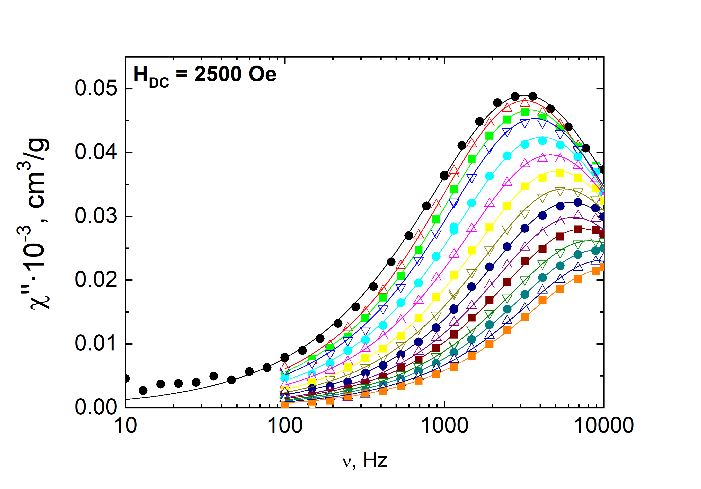 |
| --- | --- |

**Figure S8.** AC frequency dependences of the real (χ', left) and imaginary (χ'', right) parts of AC susceptibility for **5E-Co** under a 2,500 Oe field at different temperatures.

| 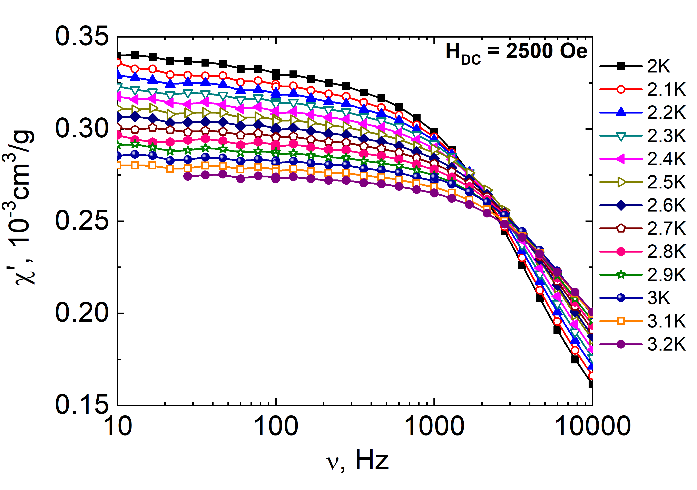 | 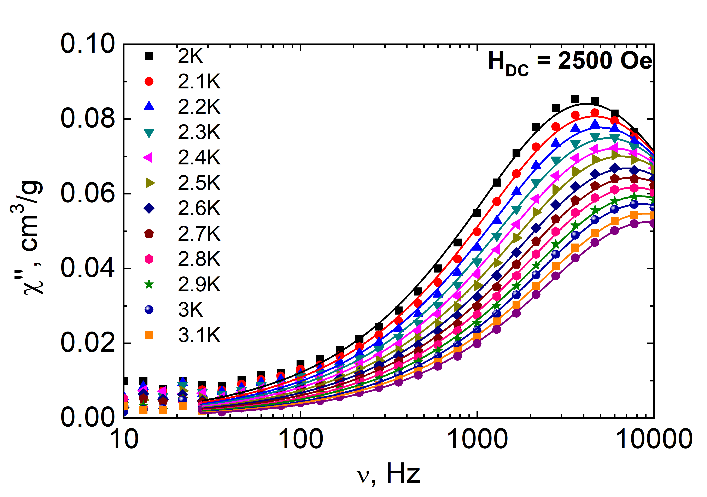 |
| --- | --- |

**Figure S9.** AC frequency dependences of the real (χ ', left) and imaginary (χ'', right) parts of AC susceptibility for **15E-Co** under a 2,500 Oe field at different temperatures.


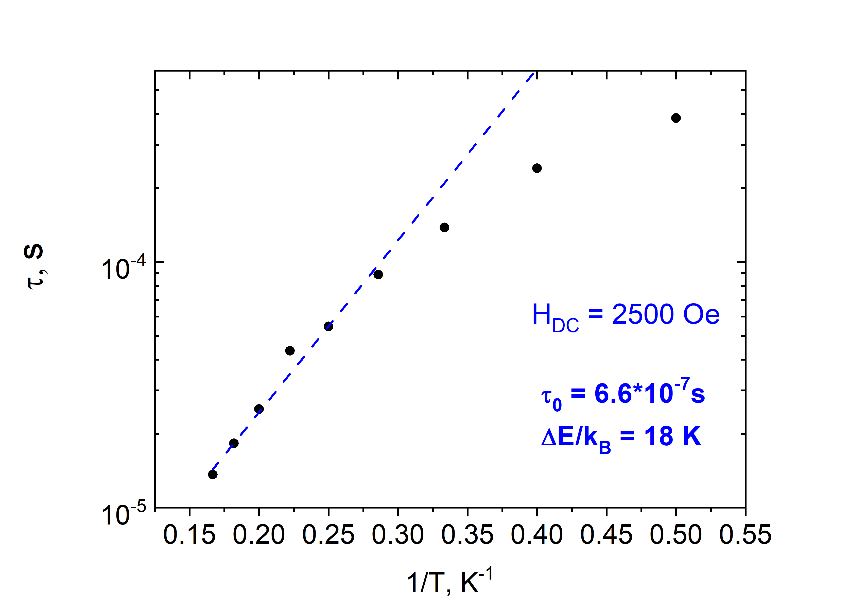


**Figure S10.** Temperature dependence of the relaxation time for **1E-Co** estimated from the generalised Debye fits of the AC susceptibility data shown in Fig. S7 collected under a 2,500 Oe field.


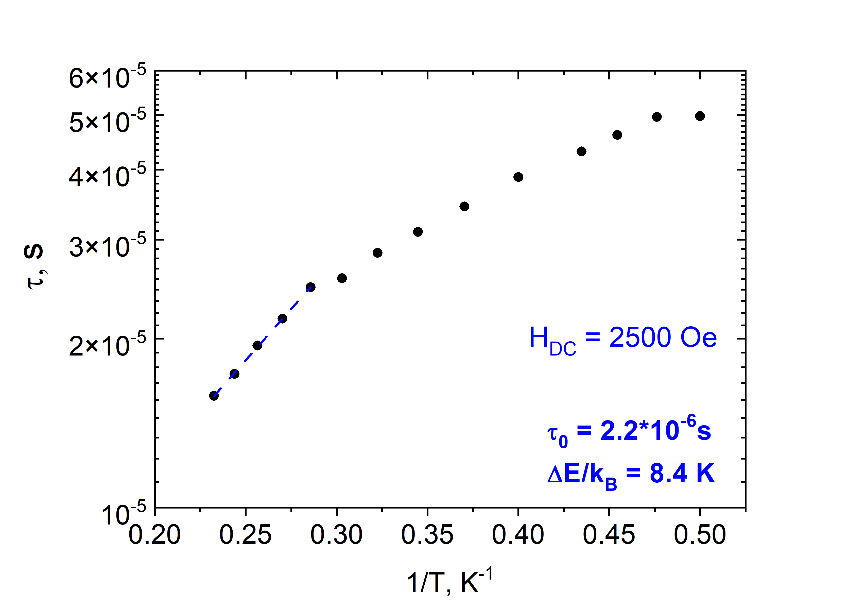


**Figure S11.** Temperature dependence of the relaxation time for **5E-Co** estimated from the generalised Debye fits of the AC susceptibility data shown in Fig. S8 collected under a 2,500 Oe field.


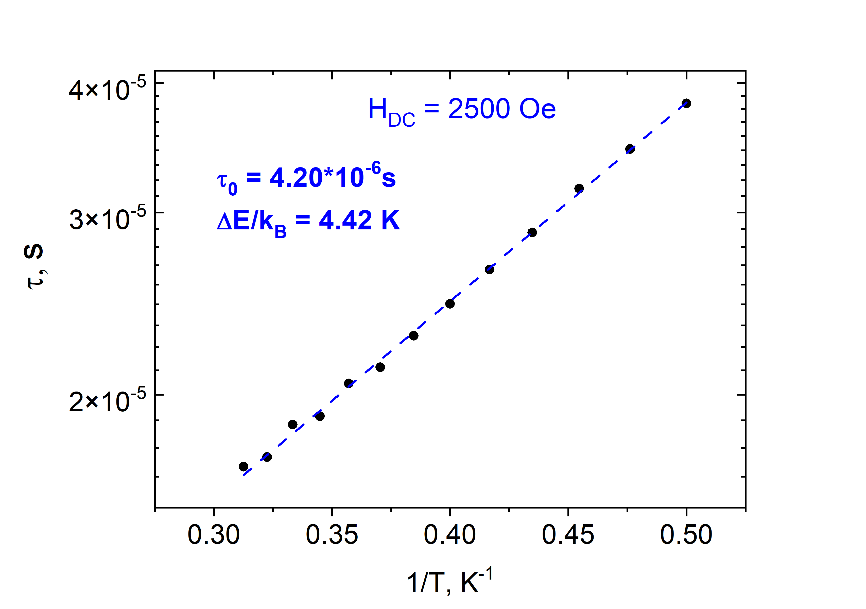


**Figure S12.** Temperature dependence of the relaxation time for **15E-Co** estimated from the generalised Debye fits of the AC susceptibility data shown in Fig. S9 collected under a 2,500 Oe field. .
